# Testing the validity of a new scale designed to assess beliefs and perceptions about colorectal cancer and colorectal cancer screening in Malaysia: a principal component analysis

**DOI:** 10.1136/bmjopen-2023-072166

**Published:** 2023-08-31

**Authors:** Tin Tin Su, Felix Oluyemi Adekunjo, Desiree Schliemann, Christopher R Cardwell, Mila Nu Nu Htay, Maznah Dahlui, Siew Yim Loh, Victoria L Champion, Michael Donnelly

**Affiliations:** 1 South East Asia Community Observatory (SEACO), Jeffery Cheah School of Medicine and Health Sciences, Monash University Malaysia, Bandar Sunway, Selangor, Malaysia; 2 Centre for Public Health and UKCRC Centre of Excellence for Public Health, Queen's University Belfast, Belfast, UK; 3 Centre for Pooulation Health (CePH), Department of Social and Preventive Medicine, Faculty of Medicine, University of Malaya, Kuala Lumpur, Malaysia; 4 Department of Economics, Faculty of Social Sciences, Lagos State University, Lagos, Nigeria; 5 Department of Community Medicine, Faculty of Medicine, Manipal University College Malaysia, Melaka, Malaysia; 6 Department of Research Development and Innovation, University Malaya Medical Centre, Kuala Lumpur, Malaysia; 7 Department of Rehabilitation Medicine, Faculty of Medicine, University of Malaya, Kuala Lumpur, Malaysia; 8 School of Nursing, Indiana University, Bloomington, Indiana, USA

**Keywords:** PUBLIC HEALTH, EPIDEMIOLOGY, Cancer pain

## Abstract

**Objective:**

To conduct a cultural adaptation and validation of the Champion Health Belief Model Scale (CHBMS) for colorectal cancer (CRC) screening (CHBMS-CRC-M) in order to assess and investigate perceptions and beliefs about CRC screening in Malaysia.

**Designs and participants:**

The results from an evidence synthesis and the outcomes from an expert panel discussion were used to shape CHBMS scale content into an assessment of beliefs about CRC screening (CHBMS-CRC). This questionnaire assessment was translated into the official language of Malaysia. An initial study tested the face validity of the new scale or questionnaire with 30 men and women from various ethnic groups. Factorial or structural validity was investigated in a community sample of 954 multiethnic Malaysians.

**Setting:**

Selangor state, Malaysia.

**Results:**

The new scale was culturally acceptable to the three main ethnic groups in Malaysia and achieved good face validity. Cronbach’s alpha coefficients ranged from 0.66 to 0.93, indicating moderate to good internal consistency. Items relating to perceived susceptibility to CRC ‘loaded’ on Factor 1 (with loadings scoring above 0.90); perceived benefits of CRC screening items loaded on factor 2 and were correlated strongly (loadings ranged between 0.63 and 0.83) and perceived barriers (PBA) to CRC screening (PBA) items loaded on factor 3 (range 0.30–0.72).

**Conclusion:**

The newly developed CHBMS-CRC-M fills an important gap by providing a robust scale with which to investigate and assess CRC screening beliefs and contribute to efforts to enhance CRC screening uptake and early detection of CRC in Malaysia and in other Malay-speaking communities in the region.

**STRENGTHS AND LIMITATIONS OF THIS STUDY**

- The development of the new Champion Health Belief Model Scale for colorectal cancer screening in Malaysia scale was informed by an evidence synthesis and iterative review by a multidisciplinary expert panel.
- We followed the procedures set out in the relevant WHO guidelines for the process of translation and adaptation of instruments and the cross-cultural survey guidelines.
- A large randomly selected sample from different ethnic groups was recruited.
- Respondents were recruited from one state in Malaysia.
- The questionnaire has not been tested for use with non-Malay-speaking ethnic groups.

## Introduction

The global burden of colorectal cancer (CRC) is increasing. Appropriate cancer control measures are needed to reduce increasing CRC mortality particularly in low-income and middle-income countries (LMICs) such as Malaysia where CRC is the second most common cancer.^1^ According to GLOBOCAN report 2020, 6597 CRC cases were detected in 2020 (13.6% of all newly diagnosed cancer cases) and CRC-related deaths rose from 2565 to 3420 within 8 years.^2 3^ It is estimated that about one-third to one-half of deaths due to CRC could be avoided through early presentation, detection and appropriate treatment.^4^ However, over 50% of CRC patients in Malaysia present at late stages.^5^ Research indicates that only 8% of people aged ≥50 years attend CRC screening, understanding about CRC screening tests is lacking^6 7^ and willingness to participate in CRC screening is low.^8^ According to Ho *et al*,^9^ about half of cancer-related deaths in Malaysia could be avoided if cancer was detected and diagnosed early. Studies indicate that negative beliefs and perceptions may play a role in late presentation and diagnosis of advanced cancer among Malaysians.^10–12^ Thus, there is a need to develop a reliable and valid measurement tool to investigate perceptions and beliefs about screening.

The health belief model (HBM) provided the theoretical framework for this study.^7^ Previous studies applied the model to breast cancer screening (BC) behaviour and, subsequently, the Champion Health Belief Model Scale (CHBMS-BC) was developed.^13^ Further refinements and revisions led initially to the addition of a subscale—confidence regarding breast self-examination^14^ and the addition of item questions to capture mammogram screening. The final revised CHBMS-BC for mammogram screening included three subscales: perceived susceptibility (PSU), benefits and barriers.^15^ The CHBMS-BC has been translated into different languages and adapted and tested in different countries^16–19^ including being used to assess the effectiveness of interventions which targeted women’s participation in BC screening programme.^20–24^ In addition, the CHBMS-BC has been adapted and validated to assess perceptions about BC screening among Malaysian women.^25 26^

As far as we are aware, a measure of the beliefs about CRC screening in Malaysia does not exist. The purpose of this study was to conduct a cultural adaptation of the CHBMS-BC questionnaire to develop a validated tool to investigate CRC screening beliefs in Malaysia.

## Method

### Evidence synthesis

We conducted a systematic review (according to PRISMA guidelines and a protocol was registered with PROSPERO) in order to synthesise the findings from Malaysia-based studies that investigated CRC symptoms awareness and barriers to cancer screening uptake.^27^

### Expert panel discussion

The CHBMS for mammogram screening was adapted for use in relation to CRC screening. An expert panel conducted the adaptation process based on the findings of the systemic review, differences in the nature of screenings and consideration of local scenarios. The panel of experts included three public health physicians, two cancer epidemiologists, one cancer advocate and one psychologist.

### Linguistic validation

The translation of the adapted scale (CHBMS-CRC-M) followed the procedures set out in the relevant WHO guidelines (WHO|Process of translation and adaptation of instruments, n.d.) and the Cross-Cultural Survey Guidelines (Adaptation—Cross-Cultural Survey Guidelines, n.d.). The questionnaire was translated into the local and official language of Malaysia (Bahasa Melayu). Two independent professional translators who are bilingual (English and Bahasa Melayu) conducted forward translation. We worked towards achieving a conceptual and culturally appropriate version rather than a literal translation. One of the researchers, who is a native Malay speaker, compared both versions and resolved any discrepancies via discussions with the translators.

### Initial testing phase

We tested the face validity of the questionnaire with 30 (male/female) participants from the major ethnic groups in Malaysia (Malay, Chinese and Indian) in order to evaluate the extent to which the questionnaire was clear, simple and easy to understand, the presence of any inappropriate, redundant, incorrectly worded or missing items and the relevancy, flow and arrangement of the questionnaire. Trained interviewers undertook face-to-face interviews. Generally, the face validity study indicated that the newly developed CHBMS-CRC-M questionnaire was culturally acceptable to ethnic Malaysian men and women.

### Main study phase

The culturally validated CHBMS-CRC-M questionnaire was administered during the baseline data collection phase of the Be Cancer Alert Campaign.^28^ The baseline survey occurred between January and March 2018 in Rawang subdistrict of the Gombak, Selangor State—the most populated state in Malaysia with a population above 5 million. Rawang subdistrict was chosen (in consultation with the Department of Statistics Malaysia: DOSM) because it contains a representative mix of ethnicities, age groups and income groups. The target sample comprised adult males and females who (1) lived in randomly selected households, (2) were above 40 years of age, (3) were fluent in Malay and (4) were able to answer independently without support from others. The age range was selected because populations in LMICs including Malaysia tend to be diagnosed with cancer at a younger age compared with high-income countries. The DOSM randomly selected 4368 houses from the 273 enumeration blocks (EBs—artificially created contiguous geographical areas with specific boundaries containing 100 households). We oversampled to take account of non-resident areas, business premises, non-respondents and survey-ineligible residents. The security guards and management offices in certain housing areas did not allow the survey to be conducted (this comprised a total of 656 houses across 36 EBs); 65 houses were non-residential business premises and 253 houses were unoccupied. The survey was conducted during official day time working hours due to our duty to ensure the safety of our interviewers. There did not appear to be residents at home in 1635 houses, most likely, because residents were working adults and were at their place of work when our interviewers called to these homes. A total of 1292 out of 1759 reachable households agreed to participate—a response rate of 73%. Finally, 954 participants who fulfilled the above-mentioned inclusion criteria completed the survey and the new CHBMS-CRC-M questionnaire. The percentage of missing data relating to items ranged from 0.4% to 2.7%.

Trained and supervised research assistants conducted face-to-face interviews after obtaining written informed consent from participants.

### Data analysis

SPSS V.22.0 statistical software was used to conduct descriptive statistics and a factor analysis (using a principal component analysis (PCA), varimax rotation, an eigenvalue of 1 and above and 0.3 as a cut-off point for loading onto a factor). Sampling adequacy was assessed using Kaiser-Meyer-Olkin (KMO), Bartlett’s test of sphericity and a KMO value of 0.70 and above.^29 30^ Reliability was assessed using Cronbach’s alpha as a test of the internal consistency of items under each component of the scale: PSU to CRC screening, perceived benefits of CRC screening (PBE) and perceived barriers to CRC screening (PBA). We interpreted Cronbach Alpha scores to indicate as follows: 0.61–0.65—moderate, 0.71–0.91—good and 0.91–0.93—strong internal consistency in keeping with Taber.^31^

### Patient and public involvement

Key stakeholders and researchers with experience in cancer prevention and control were consulted at various stages including the cultural adaptation phase and during face validity testing of the new scale. The newly developed CHBMS-CRC-M will be posted on the research team’s website (http://www.becanceralert.com/research/bcac/) and will be available freely for use by researchers and practitioners.

## Results

### Evidence synthesis

Nine studies met review eligibility criteria but only three studies included data about barriers to CRC screening uptake. Each one of the three studies used a different self-developed questionnaire to assess barriers to screening and one study included data about domains related to the HBM in terms of PSU and benefits of screening.^6 32 33^ ‘Fear of result’ from colorectal screening was the most commonly reported barrier in the three studies. Two studies reported the following comments in common which potentially could act as barriers or inhibiting factors: ‘screening is embarrassing’ (35% and 55%, respectively), ‘did not have symptoms of CRC’ (13% and 39%, respectively) and ‘FOBT was not recommended by doctor’ (11% and 35%, respectively).^30 31^ Similarly, a further two studies from the review documented that ‘fear of discomfort’ during CRC screening (30% and 64%, respectively) and ‘CRC screening is expensive’ (23% and 53%, respectively).^6 33^

### Expert panel discussion

The expert panel used the three subscales (susceptibility, benefits and barriers) of the original scale to produce the 18-item CHBMS-CRC-M. A comparison of the item content of the original CHBMS and the newly adapted and developed CHBMS-CRC-M is presented in table 1. For example, the PBA subscale to CRC screening comprised 11 items which covered emotional, practical, service and financial barriers. The response format to every item was a 5-point Likert scale: (1) represented ‘strongly disagree’ while (5) represented strongly agree. Scores on each domain or subscale ranged as follows: susceptibility (3–15), benefits (4–20) and barriers (11–55). The explanatory note about CRC screening read: ‘CRC screening is carried out to test whether or not someone has cancer. CRC can be detected through an examination of a stool and/or by examining a patient’s colon and rectum with a camera that is inserted through their posterior or backside.’

**Table 1:** Comparison of items from the original CHBMS for mammogram screening and newly developed CHBMS-CRC-M

Original CHBMS for mammogram screening	CHBMS_CRC-M
Susceptibility	
1. It is likely that I will get breast cancer.	1. It is likely that I will get colorectal cancer.
2. My chances of getting breast cancer in the next few years are great.	2. My chances of getting colorectal cancer in the next few years are high.
3. I feel I will get breast cancer sometime during my life.	3. I feel I will get colorectal cancer sometime during my life.
Benefits	
1. If I get a mammogram and nothing is found, I do not worry as much about breast cancer.	1. If I get colorectal cancer screening and nothing is found, I will worry less about colorectal cancer.
2. Having a mammogram will help me find breast lumps early.	2. Having colorectal cancer screening will help me find colorectal cancer early.
3. If I find a lump through a mammogram, my treatment for breast cancer may not be as bad.	3. If I find abnormalities through colorectal cancer screening my treatment for colorectal cancer may not be as bad.
4. Having a mammogram is the best way for me to find a very small lump.	
5. Having a mammogram will decrease my chances of dying from breast cancer.	4. Having colorectal cancer screening will decrease my chances of dying from colorectal cancer.
Barriers	
1. I am afraid to have a mammogram because I might find out something is wrong.	1. I am afraid to have colorectal cancer screening because I might find out something is wrong.
2. I am afraid to have a mammogram because I don’t understand what will be done.	2. I am afraid to have colorectal cancer screening because I don’t understand what will be done.
3. I don’t know how to go about getting a mammogram.	3. I don’t know how to go about getting colorectal cancer screening.
4. Having a mammogram is too embarrassing.	4. Having colorectal cancer screening is too embarrassing.
5. Having a mammogram takes too much time.	5. Having colorectal cancer screening takes too much time.
6. Having a mammogram is too painful.	6. Having colorectal cancer screening is too painful.
7. People doing mammograms are rude to women.	7. People doing colorectal cancer screening are rude to patients.
8. Having a mammogram exposes me to unnecessary radiation.	
9. I cannot remember to schedule a mammogram.	
10. I have other problems more important than getting a mammogram.	8. I have other problems more important than getting colorectal cancer screening.
11. I am too old to need a routine mammogram.	9. I am not the right age to need a routine colorectal cancer screening.
	10. I cannot afford to get colorectal cancer screening.
	11. I don’t have the encouragement I need from my close relatives to attend colorectal cancer screening.

CHBMS-CRC-M, Champion Health Belief Model Scale for colorectal cancer screening in Malaysia.

The final data analysis comprised a sample of 954 survey participants. Table 2 presents descriptive statistics for the sample. For example, 62% were male, 82% were married, around a quarter had at least above certificate education level; mean age was 50.84 years (SD=16.85) and, on average, there were five residents per household (SD=2.28).

**Table 2:** Sociodemographic characteristics (n=954)

Characteristic	No	Percentage
Gender		
Male	361	38
Female	593	62
Nationality		
Malaysian	871	91
Non-Malaysian	80	8
Marital status		
Single	31	3
Married	783	82
Divorcee and widow	136	15
Religion		
Islam	585	61
Christianity	35	4
Buddhism	95	10
Hinduism	226	24
Others	11	1.1
Ethnicity		
Malay	516	54
Chinese	110	12
Indian	264	28
Others	64	6.7
Education		
No formal education	73	8
Never completed primary school	79	8
Completed primary school	190	20
Completed secondary school	386	41
Certificate A-Level/STPM/HSC	99	10
Tertiary education	125	13
Current job		
Civil servant	26	3
Private sector employee	194	20
Self-employed	128	13
Retiree	116	12
Homemakers	377	40
Unemployed	113	12
Main occupation		
Manager	13	1
Professionals	50	5
Technicians and associate professionals	31	3
Clerical support workers	20	2
Service and sales workers	110	12
Skilled agricultural, forestry and fishery workers	6	0.6
Craft and related trades workers	5	0.5
Plant and machine-operators and assemblers	19	2
Elementary occupations	87	9
Healthcare professional (ie, doctors, nurses, etc)	5	0.5
Monthly family income		
Below RM2000	342	36
RM2000–RM3000	201	21
RM3000–RM4000	118	12
Above RM4000	147	16

### Principal component analysis

The sample size (n=954) in relation to the new 18-item scale and 5-point Likert response was more than required in order to conduct a robust PCA.^34^ A PCA was conducted on the 18 items of the CHBMS with varimax rotation. We applied a PCA to obtain the component or factor solution based on the linear combination of observed items.^35^

Varimax rotation was used to clarify the relationships between components and items.^36^ Researchers applied PCA and varimax rotation during the validation of the original CHBMS for BC screening,^20^ and the translated Malay version of CHBMS-BC-M.^37^ We followed a similar analytic process to the process that was used in the previously published articles (referenced above) in keeping with the original theoretically informed investigative approach to BC and mammogram screening. A key aim of the analysis was to test the appropriateness and acceptability of the adapted and supplemented item content in the context of a very different type of cancer from BC; and to conduct a PCA to investigate the nature and degree to which the item content grouped together to represent or explain beliefs about CRC and potential use of CRC screening. Ideally, further field testing would occur and at some later stage a confirmatory factor analysis might be undertaken.

The KMO test verified sampling adequacy for the conduct of a PCA (KMO=0.73) and Bartlett’s test of sphericity (χ^2^=5093.73) confirmed that the sample size was adequate. The analysis obtained eigenvalues for the factors each of which exceeded Kaiser’s criterion of 1 and in combination explained 44% of variance (table 3).

**Table 3:** Total variance explained

Item	Initial eigenvalues			Extraction sums of squared loadings			Rotation sums of squared loadings		
	Total	% of variance	Cumulative %	Total	% of variance	Cumulative %	Total	% of variance	Cumulative %
1	2.966	16.476	16.476	2.966	16.476	16.476	2.685	14.914	14.914
2	2.682	14.898	31.374	2.682	14.898	31.374	2.672	14.842	29.756
3	2.182	12.123	43.497	2.182	12.123	43.497	2.473	13.740	43.497
4	1.393	7.738	51.234						
5	1.089	6.048	57.282						
6	1.046	5.810	63.092						
7	0.923	5.130	68.222						
8	0.874	4.856	73.078						
9	0.777	4.317	77.395						
10	0.731	4.059	81.453						
11	0.643	3.573	85.027						
12	0.632	3.514	88.540						
13	0.569	3.159	91.700						
14	0.474	2.631	94.331						
15	0.380	2.112	96.443						
16	0.278	1.547	97.990						
17	0.250	1.388	99.379						
18	0.112	0.621	100.000						

Items relating to PSU to CRC ‘loaded’ on factor 1 (with loadings above 0.90); PBE of CRC screening items loaded on factor 2 and were correlated strongly (loadings ranged between 0.63 and 0.83); and PBA to CRC screening items loaded on factor 3 (range 0.30–0.72). Table 4 presents the rotated factor matrix and three-factor solution (items greater than 0.3 were retained as recommended by best practice).^30^

**Table 4:** CHBMS-CRC-M item loadings for each factor

Subscales and items	Factor loadings	Factor loadings	Factor loadings
Susceptibility			
PSU1	0.944		
PSU2	0.943		
PSU3	0.903		
Benefits			
PBE1		0.627	
PBE2		0.826	
PBE3		0.799	
PBE4		0.760	
Barriers			
PBA1			0.716
PBA2			0.702
PBA3			0.305
PBA4			0.619
PBA5			0.391
PBA6			0.429
PBA7			0.428
PBA8			0.533
PBA9			0.419
PBA10			0.304
PBA11			0.321

CHBMS-CRC-M, Champion Health Belief Model Scale for colorectal cancer screening in Malaysia; PBA, perceived barriers; PBE, perceived benefits; PSU, perceived susceptibility.

### Internal reliability or consistency

The PSU to CRC indicated high reliability (Cronbach’s α=0.93, with all items strongly correlated around 0.80); the PBE of CRC screening (PBE) had fairly high overall internal consistency (Cronbach’s α=0.78 though inter-item correlations ranged from 0.31 to 0.57); and the reliability test for PBA to CRC screening was moderate (Cronbach’s α=0.66 with some inter-item correlations below 0.30).

## Discussion

Although annual CRC screening is recommended for people aged ≥50 years in Malaysia,^38^ a population-based screening programme is not in place and efforts so far to improve uptake have been selective and sporadic.^39^ The absence of a population-based screening programme and the development of advanced cancer impacts negatively on survival,^5 40^ requires more intensive treatment, increases healthcare resource utilisation and places additional financial burden on households. The healthcare system and context in Malaysia requires systematic research attention in order to ensure the successful implementation of CRC screening, particularly in terms of the promotion, acceptance of and participation in, CRC screening among the general population. Misperception of risk of cancer, denial of the existence of the disease, emotional barriers and negative beliefs about cancer screening generally affect screening uptake and lead to delay in seeking medical help.

An in-depth understanding about how adults perceive their susceptibility to CRC, the benefits of attending screening and barriers to CRC screening is crucial for the successful implementation of CRC screening and enhancement of screening coverage. A systematic review comprising 30 articles evaluated the association between HBM domains and CRC screening in the community.^41^ The review found that higher scores for the domains, PSU and benefits, and lower scores for the barriers domain were associated with positive screening behaviour; and cues to action were associated with CRC screening adherence.^41^ The results of the review provide support for the potential benefits of using the new scale in terms of improved understanding and providing insights into the design of screening uptake interventions.

We adapted, translated and tested the CHBMS-CRC-M in order to create a means of evaluating Malaysians’ beliefs or perceptions about CRC screening. The cultural adaptation of the CHBMS-CRC-M was grounded robustly via an evidence synthesis, an expert panel discussion and linguistic validation exercise. The scale was proven to have good face validity in a relatively small separate survey from the main study which assessed its structural validity in a large randomly selected community sample of multiethnic Malay-speaking men and women.

The results of our validation study showed that the newly developed CHBMS-CRC-M is a reliable and valid instrument for measuring beliefs regarding CRC screening among Malay-speaking multiethnic Malaysians. Cronbach’s alpha coefficients for the three subscales ranged from 0.66 to 0.93, which indicated moderate to high internal consistency. The PCA revealed that each item of the scale showed acceptable item correlations (greater than the criterion of 0.30). The relevant items from the 18-item pool loaded separately and sufficiently onto three factors or components in a pattern and manner that was consistent with the three subscales of PSU to CRC, PBE of CRC screening and PBA to CRC screening. Barriers tend to vary in nature (eg, emotional, practical, service and financial barriers) and a given barrier may have more, less or the same perceived salience for different respondents depending on a range of psychosocial health and life experiences (with resultant implications for intervention design and delivery). Arguably, given this point, it is surprising that a Cronbach’s alpha for PBA domain of 0.66 was achieved although less than the convention of <0.7. We interpreted 0.6 as indicating an acceptable moderate level of internal reliability (in keeping with other researchers, eg, Taber, 2018) and viewed the PBA domain to be a single discrete domain that was consistent with the HBM.^20^

The new measure appears to have good structural and construct validity for the assessment of beliefs about CRC and CRC screening as well as good face validity and internal reliability. Our findings are in broad agreement with a study in Korea that translated and validated the CHBMS for CRC screening.^42^ However, the analysis of the much longer 45-item Korean version generated an additional domain or component (in addition to the above three components) that assessed beliefs related to personal self-efficacy. In contrast, our study led to a shorter version of CHBMS that was adapted, translated and validated to be applicable in the Malay-speaking community. We recognise the long-established importance of self-efficacious beliefs to performing positive health behaviours and avoiding harmful behaviours and, so, researchers may wish to avail of the option of adding one of the many existing short, easy to use well-validated self-efficacy scales for cancer screening though there is a need to be mindful of respondent burden and keeping respondents engaged sufficiently to achieve the goal of CRC screening uptake.

The newly developed CHBMS-CRC-M fills an important gap by providing a robust scale with which to investigate and assess CRC screening beliefs and, so, contribute to the development and implementation of culturally sensitive and effective programmes that will enhance CRC screening uptake and early detection of CRC in Malaysia. This scale is suitable, too, for use with other Malay-speaking communities in the region such as Indonesia, Singapore, Philippine, southern Thailand and southern Myanmar.

### Strengths and limitations

This study applied a thorough research-informed validation process to develop the new CHBMS-CRC-M scale. The reasonably large and randomly selected sample from the different ethnic groups was recruited to ascertain cultural acceptability and applicability across the multiethnic Malay-speaking people in Malaysia. However, it is important to note that respondents were recruited from Selangor State in Malaysia, and the scale/questionnaire has not been tested with non-Mala-speaking ethnic groups.

## Conclusion

There is an urgent need to improve CRC screening uptake in Malaysia and efforts are needed to address negative beliefs and barriers to CRC screening. The newly developed CHBMS-CRC-M is a reliable and valid scale to measure the beliefs on CRC screening in Malay-speaking communities in Malaysia. In addition to using the scale in public health research and service development, the information gathered by using CHBMS-CRC-M may prove to be useful for primary care physicians and staff in terms of helping to identify and reduce misperceptions and barriers to cancer screening, thereby facilitating attendance and improving uptake.

## Supplementary Material

Reviewer comments

Author's manuscript

## Data Availability

Data are available on reasonable request. Data are available from the corresponding authors, MDo and TTS, on reasonable request.
