# Supplementary material for: Testing the validity of a new scale designed to assess beliefs and perceptions about colorectal cancer and colorectal cancer screening in Malaysia: a principal component analysis
Source: BMJ Open. 2023 Aug 31;13(8):e072166. doi: 10.1136/bmjopen-2023-072166 (PMC10476116; doi:10.1136/bmjopen-2023-072166)
Supplement: Author's manuscript [file bmjopen-2023-072166.draft_revisions.pdf]

BMJ Open is committed to open peer review. As part of this commitment we make the peer review history of every article we publish publicly available.

When an article is published we post the peer reviewers' comments and the authors' responses online. We also post the versions of the paper that were used during peer review. These are the versions that the peer review comments apply to.

The versions of the paper that follow are the versions that were submitted during the peer review process. They are not the versions of record or the final published versions. They should not be cited or distributed as the published version of this manuscript.

BMJ Open is an open access journal and the full, final, typeset and author-corrected version of record of the manuscript is available on our site with no access controls, subscription charges or pay-per-view fees (http://bmjopen.bmj.com).

If you have any questions on BMJ Open's open peer review process please email info.bmjopen@bmj.com

BMJ Open

Testing the validity of a new scale designed to assess beliefs and perceptions about colorectal cancer and colorectal cancer screening in Malaysia: A principal component analysis

Journal:	*BMJ Open*
Manuscript ID	bmjopen-2023-072166
Article Type:	Original research
Date Submitted by the Author:	02-Feb-2023

Complete List of Authors:	Su, Tin ; Monash University - Malaysia Campus, Jeffrey Cheah School of Medicine and Health Sciences<br>Adekunjo, Felix ; Lagos State University, Department of Economics, Faculty of Social Sciences<br>Schliemann, Desiree ; Queen's University Belfast, Centre for Public Health<br>Cardwell, Christopher; Queen's University Belfast, School of Medicine, Dentistry and Biomedical Sciences<br>Htay , Mila; University of Malaya, Department of Social and Preventive Medicine<br>Dahlui, Maznah; University of Malaya, Department of Social and Preventive Medicine<br>Loh, Siew_Yim; University Malaya, Rehabilitation<br>Champion, Victoria; Indiana University, School of Nursing<br>Donnelly, Michael; Queen's University Belfast, Centre for Public Health

Keywords:	PUBLIC HEALTH, EPIDEMIOLOGY, Cancer pain < ONCOLOGY

SCHOLARONE™
Manuscripts

BMJ

I, the Submitting Author has the right to grant and does grant on behalf of all authors of the Work (as defined in the below author licence), an exclusive licence and/or a non-exclusive licence for contributions from authors who are: i) UK Crown employees; ii) where BMJ has agreed a CC-BY licence shall apply, and/or iii) in accordance with the terms applicable for US Federal Government officers or employees acting as part of their official duties; on a worldwide, perpetual, irrevocable, royalty-free basis to BMJ Publishing Group Ltd ("BMJ") its licensees and where the relevant Journal is co-owned by BMJ to the co-owners of the Journal, to publish the Work in this journal and any other BMJ products and to exploit all rights, as set out in our licence.

The Submitting Author accepts and understands that any supply made under these terms is made by BMJ to the Submitting Author unless you are acting as an employee on behalf of your employer or a postgraduate student of an affiliated institution which is paying any applicable article publishing charge ("APC") for Open Access articles. Where the Submitting Author wishes to make the Work available on an Open Access basis (and intends to pay the relevant APC), the terms of reuse of such Open Access shall be governed by a Creative Commons licence – details of these licences and which Creative Commons licence will apply to this Work are set out in our licence referred to above.

Other than as permitted in any relevant BMJ Author's Self Archiving Policies, I confirm this Work has not been accepted for publication elsewhere, is not being considered for publication elsewhere and does not duplicate material already published. I confirm all authors consent to publication of this Work and authorise the granting of this licence.

For peer review only

Testing the validity of a new scale designed to assess beliefs and perceptions about colorectal cancer and colorectal cancer screening in Malaysia: A principal component analysis

Tin Tin Su[1,2, 4*], Felix Oluyemi Adekunjo[3], Désirée Schliemann[4], Christopher Cardwell[4], Mila Nu Nu Htay[2,5], Maznah Dahlui[2], Siew Yim Loh[6] , Victoria Champion[7] and Michael Donnelly[4]

Affiliation:

1) South East Asia Community Observatory (SEACO) & Global Public Health, Jeffery Cheah School of Medicine and Health Sciences, Monash University Malaysia, Bandar Sunway, Malaysia

2) Centre for Population Health (CePH), Department of Social and Preventive Medicine, Faculty of Medicine, University of Malaya, Kuala Lumpur, Malaysia

3) Department of Economics, Faculty of Social Sciences, Lagos State University, Lagos, Nigeria

4) Centre for Public Health and UKCRC Centre of Excellence for Public Health, Queen's University Belfast, UK

5) Department of Community Medicine, Faculty of Medicine, Manipal University College Malaysia, Jalan Batu Hampar, 75150 Bukit Baru, Melaka, Malaysia

6) Department of Rehabilitation Medicine, Faculty of Medicine, University of Malaya, Kuala Lumpur, Malaysia

7) Indiana University, School of Nursing, USA

*Tin Tin Su (Corresponding author)

Affiliation: South East Asia Community Observatory (SEACO) & Global Public Health, Jeffery Cheah School of Medicine and Health Sciences, Monash University Malaysia, Bandar Sunway, Malaysia

Department of Social and Preventive Medicine, University of Malaya, Kuala Lumpur, Malaysia

Phone: +603-5514 447

For peer review only

*** TinTin.Su@monash.edu**

Running title: A questionnaire to assess beliefs about colorectal cancer and screening

For peer review only

For peer review only - http://bmjopen.bmj.com/site/about/guidelines.xhtml

Abstract

Objective: To conduct a cultural adaptation and validation of the Champion Health Belief Model Scale (CHBMS) for colorectal (CRC) cancer screening (CHBMS-CRC-M) in order to assess and investigate perceptions and belief about CRC screening in Malaysia.

Designs and participants: The results from an evidence synthesis and the outcomes from an expert panel discussion were used to shape CHBMS scale content into an assessment of beliefs about CRC screening (CHBMS-CRC) which was translated into the official language of Malaysia. An initial study tested the face validity of the new scale with 30 men and women from various ethnic groups. Factorial or structural validity was investigated in a community sample of 954 multi-ethnic Malaysians.

Setting: Selangor state, Malaysia

Results: The new scale was culturally acceptable to the ethnic groups and achieved good face validity. Cronbach's alpha coefficients ranged from 0.66 to 0.93 indicating moderate to good internal consistency. Items relating to perceived susceptibility to CRC (PSU) 'loaded' on Factor 1 (with loadings above 0.90); perceived benefits of CRC screening (PBE) items loaded on Factor 2 and were correlated strongly (loadings ranged between 0.63 and 0.83); and perceived barriers to CRC screening (PBA) items loaded on Factor 3 (range: 0.30 to 0.72).

Conclusion: The newly developed CHBMS-CRC-M fills an important gap by providing a robust scale with which to investigate and assess CRC screening beliefs and, so, contribute to efforts to enhance CRC screening uptake and early detection of CRC in Malaysia and other Malay speaking communities in the region.

Keywords: Beliefs, colorectal cancer, cancer screening, questionnaire validation, cultural adaptation, Malaysia

For peer review only - http://bmjopen.bmj.com/site/about/guidelines.xhtml

Strengths and Limitations

- The development of the new CHBMS-CRC-M scale was informed by an evidence synthesis and iterative review by an expert multidisciplinary panel
- We followed the procedures set out in the relevant WHO guidelines for the Process of translation and adaptation of instruments and the Cross-Cultural Survey Guidelines
- A large randomly selected sample from different ethnic groups was recruited
- Respondents were recruited from one state in Malaysia
- The questionnaire has not been tested for use with non-Malay speaking ethnic groups

For peer review only

For peer review only - http://bmjopen.bmj.com/site/about/guidelines.xhtml

Introduction

The global burden of colorectal cancer (CRC) is increasing. Appropriate cancer control measures are needed to reduce increasing CRC mortality particularly in LMICs such as Malaysia where CRC is the second most common cancer[1]. According to GLOBOCAN report 2020, 6,597 CRC cases were detected in 2020 (13.6% of all newly diagnosed cancer cases) and CRC-related deaths rose from 2,565 to 3,420 within 8 years. [2,3]. It is estimated that about one-third to one-half of deaths due to CRC could be avoided through early presentation, detection and appropriate treatment[4]. However, over 50% of CRC patients in Malaysia present at late stages[5]. Research indicates that only 8% of people aged ≥50 years attend CRC screening, understanding about CRC screening tests is lacking[6 7] and willingness to participate in CRC screening is low[8]. According to Ho, et al. [9], about half of cancer-related deaths in Malaysia could be avoided if cancer was detected and diagnosed early. Studies indicate that negative beliefs and perceptions may play a role in late presentation and diagnosis of advanced cancer among Malaysians[10-12]. Thus, there is a need to develop a reliable and valid measurement tool with which to investigate perceptions and beliefs about screening.

The Health Belief Model (HBM) provided the theoretical framework for this study[7]. Previous studies applied the model to breast screening (BC) behaviour and, subsequently, the Champion Health Belief Model Scale (CHBMS-BC) was developed[13]. Further refinements and revisions led initially to the addition of a subscale - confidence regarding breast self-examination[14] and the addition of item questions to capture mammogram screening. The final revised CHBMS-BC for mammogram screening included three subscales: perceived susceptibility, benefits and barriers[15]. The CHBMS-BC has been translated into different languages and adapted and tested in different countries[16-19] including being used to assess the effectiveness of interventions which targeted women's participation in BC screening programme[20-24]. In addition, the

For peer review only - http://bmjopen.bmj.com/site/about/guidelines.xhtml

CHBMS-BC has been adapted and validated to assess perceptions about BC screening among Malaysian women[25 26].

As far as we are aware, a measure of the beliefs about colorectal cancer screening in Malaysia does not exist. The purpose of this study was to conduct a cultural adaptation of the CHBMS-BC questionnaire to develop a validated tool to investigate CRC screening beliefs in Malaysia

Method

Evidence synthesis

We conducted a systematic review (according to PRISMA guidelines and a protocol was registered with PROSPERO) in order to synthesize the findings from Malaysia-based studies that investigated CRC symptoms awareness and barriers to cancer screening uptake[27].

Expert panel discussion

The CHBMS for mammogram screening was adapted for use in relation to CRC screening. An expert panel conducted the adaptation process based on the findings of the systemic review, differences in the nature of screenings and consideration of local scenarios. The panel of experts included three public health physicians, two cancer epidemiologists, one cancer advocate and one psychologist.

Linguistic validation

The translation of the adapted scale (CHBMS-CRC-M) followed the procedures set out in the relevant WHO guidelines (*WHO | Process of translation and adaptation of instruments*, n.d.) and the Cross-Cultural Survey Guidelines (CCSG) (*Adaptation—Cross-Cultural Survey Guidelines*, n.d.). The questionnaire was translated into the local and official language of Malaysia (Bahasa Melayu). Two independent professional translators who are bilingual (English and Bahasa Melayu) conducted forward translation. We worked towards achieving a conceptual and culturally appropriate version rather than a literal translation. One of the

For peer review only

researchers, who is a native Malay speaker, compared both versions and resolved any discrepancies via discussions with the translators.

Initial testing phase

We tested the face validity of the questionnaire with 30 (male/female) participants from the major ethnic groups in Malaysia (Malay, Chinese & Indian) in order to evaluate the extent to which the questionnaire was clear, simple and easy to understand, the presence of any inappropriate, redundant, incorrectly worded or missing items and the relevancy, flow and arrangement of the questionnaire. Trained interviewers undertook face-to-face interviews. Generally, the face validity study indicated that the newly developed CHBMS-CRC-M questionnaire was culturally acceptable to ethnic Malaysian men and women.

Main study phase

The culturally validated CHBMS-CRC-M questionnaire was administered during the baseline data collection phase of the *Be Cancer Alert Campaign* (BCAC) [28]. The baseline survey occurred between January and March 2018 in Rawang sub-district of the Gombak, Selangor State - the most populated state in Malaysia with a population above 5 million. Rawang sub-district was chosen (in consultation with the Department of Statistics: DOSM) because it contains a representative mix of ethnicities, age groups and income groups. The target sample comprised adult males and females who 1) lived in randomly selected households, 2) were above 40 years of age, 3) were fluent in Malay and 4) were able to answer independently without support from others. The age-range was selected because populations in low-middle-income countries (LMIC) including Malaysia tend to be diagnosed with cancer at a younger age compared to high income countries. The DOSM randomly selected 4,368 houses from the 273 Enumeration Blocks (EBs - artificially created contiguous geographical areas with specific boundaries containing 100 households). We oversampled to take account of non-resident areas, business premises, non-respondents and survey-ineligible residents. The

For peer review only - http://bmjopen.bmj.com/site/about/guidelines.xhtml

security guards and management offices did not allow the survey to be conducted in housing areas, which comprised a total of 656 houses across 36 EBs. 65 houses were non-residential business premises, and 253 houses were unoccupied. The survey was conducted during official day time working hours due to our duty to ensure the safety of our interviewers. There did not appear to be residents at home in 1,635 houses, most likely, because residents were working adults and were at their place of work when our interviewers called to these homes. A total of 1,292 out of 1,759 reachable households agreed to participate a response rate of 73%. Finally, 954 participants who fulfilled the above-mentioned inclusion criteria completed the survey and new CHBMS-CRC-M questionnaire. The percentage of missing data relating to items ranged from (0.4 – 2.7 %).

Trained and supervised research assistants conducted face-to-face interviews after obtaining written informed consent from participants.

Data analysis

SPSS v22.0 statistical software was used to conduct descriptive statistics and a factor analysis (using a principle component analysis, varimax rotation, an eigenvalue of 1 and above, and measurement items loadings of 0.3 as a cut-off point for loading onto a factor). Sampling adequacy was assessed using Kaiser-Meyer-Olkin (KMO), Bartlett's test of sphericity and a KMO value of 0.70 and above [29 30]. Reliability was assessed using Cronbach's Alpha as a test of the internal consistency of items under each component of the scale: perceived susceptibility to CRC screening (PSU), perceived benefits of CRC screening (PBE) and perceived barriers to CRC screening (PBA). We interpreted Cronbach Alpha scores to indicate as follows: 0.61-0.65 - moderate, 0.71-0.91 - good, and 0.91-0.93 - strong internal consistency in keeping with Taber (2018) [31].

For peer review only - http://bmjopen.bmj.com/site/about/guidelines.xhtml

Patient and Public Involvement

Key stakeholders and researchers with experience in cancer prevention and control were consulted at various stages including the cultural adaptation phase and during face validity testing of the new scale. The newly developed CHBMS-CRC-M will be posted on the research team's website and will be available freely for use by researchers and practitioners.

Results

Evidence synthesis

Nine studies met review eligibility criteria but only three studies included data about barriers to CRC screening uptake. Each one of the three studies used a different self-developed questionnaire to assess barriers to screening and one study included data about domains related to the HBM in terms of perceived susceptibility and benefits of screening[6 32 33]. "Fear of result" from colorectal screening was the most commonly reported barrier in the three studies. Two studies reported the following comments in common which potentially could act as barriers or inhibiting factors: "screening is embarrassing" (35% and 55% respectively), "did not have symptoms of CRC" (13% and 39% respectively) and "FOBT was not recommended by doctor" (11% and 35% respectively)[30 31]. Similarly, a further two studies from the review documented that "fear of discomfort" during CRC screening (30% and 64% respectively) and "CRC screening is expensive" (23% and 53% respectively) [6 33].

Expert panel discussion

The expert panel used the three subscales (susceptibility, benefits, and barriers) of the original scale to produce the 18-item CHBMS-CRC-M. A comparison of the item content of the original CHBMS and the newly adapted and developed CHBMS-CRC-M is presented in Table 1. For example, the perceived barriers subscale to colorectal cancer screening comprised 11 items which covered emotional, practical, service and financial barriers. The response format to every item was a 5-point Likert Scale: (1) represented 'strongly disagree' while (5) represented

For peer review only

strongly agree. Scores on each domain or subscale ranged as follows: susceptibility (3-15), benefits (4-20) and barriers (11-55). The explanatory note about CRC screening read: *"Colorectal cancer screening is carried out to test whether or not someone has cancer. Colorectal cancer can be detected through an examination of a stool and/or by examining colon and rectum with a camera that is put in through the backside."*

For peer review only

For peer review only - http://bmjopen.bmj.com/site/about/guidelines.xhtml

Table.1 Comparison of items from the original CHBMS for mammogram screening and newly developed CHBMS-CRC-M

Original CHBMS for mammogram screening	CHBMS_CRC-M
Susceptibility	
1. It is likely that I will get breast cancer.	1. It is likely that I will get colorectal cancer.
2. My chances of getting breast cancer in the next few years are great.	2. My chances of getting colorectal cancer in the next few years are high.
3. I feel I will get breast cancer sometime during my life.	3. I feel I will get colorectal cancer sometime during my life.
Benefits	
1. If I get a mammogram and nothing is found, I do not worry as much about breast cancer.	1. If I get colorectal cancer screening and nothing is found, I will worry less about colorectal cancer.
2. Having a mammogram will help me find breast lumps early.	2. Having colorectal cancer screening will help me find colorectal cancer early.
3. If I find a lump through a mammogram, my treatment for breast cancer may not be as bad.	3. If I find abnormalities through colorectal cancer screening my treatment for colorectal cancer may not be as bad.
4. Having a mammogram is the best way for me to find a very small lump.	
5. Having a mammogram will decrease my chances of dying from breast cancer.	4. Having colorectal cancer screening will decrease my chances of dying from colorectal cancer.
Barriers	
1. I am afraid to have a mammogram because I might find out something is wrong.	1. I am afraid to have colorectal cancer screening because I might find out something is wrong.
2. I am afraid to have a mammogram because I don't understand what will be done.	2. I am afraid to have colorectal cancer screening because I don't understand what will be done.
3. I don't know how to go about getting a mammogram.	3. I don't know how to go about getting colorectal cancer screening.
4. Having a mammogram is too embarrassing.	4. Having colorectal cancer screening is too embarrassing.
5. Having a mammogram takes too much time.	5. Having colorectal cancer screening takes too much time.
6. Having a mammogram is too painful.	6. Having colorectal cancer screening is too painful.
7. People doing mammograms are rude to women.	7. People doing colorectal cancer screening are rude to patients.
8. Having a mammogram exposes me to unnecessary radiation.	
9. I cannot remember to schedule a mammogram.	
10. I have other problems more important than getting a mammogram.	8. I have other problems more important than getting colorectal cancer screening.
11. I am too old to need a routine mammogram.	9. I am not the right age to need a routine colorectal cancer screening.
	10. I cannot afford to get colorectal cancer screening.
	11. I don't have the encouragement I need from my close relatives to attend colorectal cancer screening.

For peer review only - http://bmjopen.bmj.com/site/about/guidelines.xhtml

Data from the 954 participants was included in the final data analysis. Table 2 presents descriptive statistics for the sample. For example, 62% were male, 82% were married around a quarter had education at least above certificate level; mean age was 50.84 years (SD = 16.85) and, on average, there were five residents per household (SD = 2.28).

Table 2: Sociodemographic characteristics (n=954)

Characteristic	Number	Percentage
**Gender**		
Male	361	38
Female	593	62
**Nationality**		
Malaysian	871	91
Non-Malaysian	80	8
**Marital Status**		
Single	31	3
Married	783	82
Divorcee & Widow	136	15
**Religion**		
Islam	585	61
Christianity	35	4
Buddhism	95	10
Hinduism	226	24
Others	11	1.1
**Ethnicity**		
Malay	516	54
Chinese	110	12
Indian	264	28
Others	64	6.7
**Education**		
No formal education	73	8
Never completed primary school	79	8
Completed primary school	190	20
Completed secondary school	386	41
Certificate A-Level/STPM/HSC	99	10
Tertiary education	125	13
**Current Job**		
Civil servant	26	3
Private sector employee	194	20
Self-employed	128	13
Retiree	116	12
Homemakers	377	40
Unemployed	113	12
**Main Occupation**		
Manager	13	1
Professionals	50	5
Technicians and associate professionals	31	3
Clerical support workers	20	2
Service and sales workers	110	12
Skilled agricultural, forestry and fishery workers	6	.6
Craft and related trades workers	5	.5
Plant and machine-operators and assemblers	19	2

For peer review only

Elementary occupations<br>Healthcare professional (i.e. doctors, nurses, etc)	87<br>5	9<br>.5
**Monthly Family Income**<br>Below RM 2, 000<br>RM 2, 000 - RM 3, 000<br>RM 3, 000 - RM 4, 000<br>Above RM 4, 000	 342<br>201<br>118<br>147	 36<br>21<br>12<br>16

Principal Component Analysis

The sample size (n=954) in relation to the new 18-item scale and 5-point Likert response was more than required in order to conduct a robust principal component analysis (PCA)[34]. A PCA was conducted on the 18 items of the CHBMS with varimax rotation. We applied (PCA) to obtain the component or factor solution based on the linear combination of observed items [35].

Varimax rotation was used to clarify the relationships between components and items[36]. Researchers applied PCA and varimax rotation during the validation of the original CHBMS for breast cancer screening [20], and the translated Malay version of CHBMS-BC-M [37]. We followed a similar analytic process to the process that was used in the previously published articles (referenced above) in keeping with the original theoretically informed investigate approach to BC and mammogram screening. A key aim of the analysis was to test the appropriateness and acceptability of the adapted and supplemented item content in the context of a very different type of cancer from BC; and to conduct a PCA to investigate the nature and degree to which the item content grouped together to represent or explain beliefs about CRC and potential use of CRC screening. Ideally, further field testing would occur and at some later stage a confirmatory factor analysis might be undertaken.

The Kaiser-Meyer-Olkin test verified sampling adequacy for the conduct of a PCA (KMO= 0.73) and Bartlett's test of sphericity (Chi-square = 5093.73) confirmed that the sample size

For peer review only - http://bmjopen.bmj.com/site/about/guidelines.xhtml

was adequate. The analysis obtained eigenvalues for the factors each of which exceeded Kaiser's criterion of 1 and in combination explained 44% of variance (Table 3).

Table 3: Total Variance Explained

Item	Initial Eigenvalues			Extraction Sums of Squared Loadings			Rotation Sums of Squared Loadings		
	Total	% of Variance	Cumulative %	Total	% of Variance	Cumulative %	Total	% of Variance	Cumulative %
1	2.966	16.476	16.476	2.966	16.476	16.476	2.685	14.914	14.914
2	2.682	14.898	31.374	2.682	14.898	31.374	2.672	14.842	29.756
3	2.182	12.123	43.497	2.182	12.123	43.497	2.473	13.740	43.497
4	1.393	7.738	51.234						
5	1.089	6.048	57.282						
6	1.046	5.810	63.092						
7	.923	5.130	68.222						
8	.874	4.856	73.078						
9	.777	4.317	77.395						
10	.731	4.059	81.453						
11	.643	3.573	85.027						
12	.632	3.514	88.540						
13	.569	3.159	91.700						
14	.474	2.631	94.331						
15	.380	2.112	96.443						
16	.278	1.547	97.990						
17	.250	1.388	99.379						
18	.112	.621	100.000						

For peer review only - http://bmjopen.bmj.com/site/about/guidelines.xhtml

Items relating to perceived susceptibility to CRC (PSU) 'loaded' on Factor 1 (with loadings above 0.90); perceived benefits of CRC screening (PBE) items loaded on Factor 2 and were correlated strongly (loadings ranged between 0.63 and 0.83); and perceived barriers to CRC screening (PBA) items loaded on Factor 3 (range: 0.30 to 0.72). Table 4 presents the rotated factor matrix and three-factor solution (items greater than 0.3 were retained as recommended by best practice) [30].

Table 4. CHBMS-CRC-M item loadings for each factor

**Subscales and items**	**Factor loadings**	**Factor loadings**	**Factor loadings**
Susceptibility			
PSU1	.944		
PSU2	.943		
PSU3	.903		
Benefits			
PBE1		.627	
PBE2		.826	
PBE3		.799	
PBE4		.760	
Barriers			
PBA1			.716
PBA2			.702
PBA3			.305
PBA4			.619
PBA5			.391
PBA6			.429
PBA7			.428
PBA8			.533
PBA9			.419
PBA10			.304
PBA11			.321

Internal reliability or consistency

The perceived susceptibility to CRC (PSU) indicated high reliability (Cronbach's α = 0.93, with all items strongly correlated around 0.80); the perceived benefits of CRC screening (PBE)

For peer review only - http://bmjopen.bmj.com/site/about/guidelines.xhtml

had fairly high overall internal consistency (Cronbach's α = 0.78 though inter-item correlations ranged from 0.31 to 0.57); and the reliability test for perceived barriers to CRC screening was moderate (Cronbach's α = 0.66 with some inter-item correlations below 0.30).

Discussion

Although annual CRC screening is recommended for people aged ≥50 years in Malaysia [38], a population-based screening programme is not in place and efforts so far to improve uptake have been selective and sporadic [39]. The absence of a population-based screening programme and the development of advanced cancer impacts negatively on survival [5 40], requires more intensive treatment, increases health care resource utilization and places additional financial burden on households. The health care system and context in Malaysia requires systematic research attention in order to ensure the successful implementation of CRC screening, particularly in terms of the promotion, acceptance of, and particpation in, CRC screening among the general population. Misperception of risk of cancer, denial of the existence of the disease, emotional barriers and negative beliefs about cancer screening generally affect screening uptake and delay in seeking medical help.

An in-depth understanding about how adults perceive their susceptibility to CRC, the benefits of attending screening and barriers to CRC screening is crucial for the successful implementation of CRC screening and enhancement of screening coverage. A systematic review comprising 30 articles evaluated the association between HBM domains and CRC screening in the community[41]. The review found that higher scores for the domains, perceived susceptibility and benefits, and lower scores for the barriers domain were associated with positive screening behaviour; and cues to action were associated with CRC screening adherence[41]. The results of the review provide support for the potential benefits of using the

For peer review only - http://bmjopen.bmj.com/site/about/guidelines.xhtml

new scale in terms of improved understanding and providing insights into the design of screening uptake interventions.

We adapted, translated, and tested the CHBMS-CRC-M in order to create a means of evaluating Malaysians' beliefs or perceptions about CRC screening. The cultural adaptation of the CHBMS-CRC-M was grounded robustly via an evidence synthesis, an expert panel discussion and linguistic validation exercise. The scale was proven to have good face validity in a relatively small separate survey from the main study which assessed its structural validity in a large randomly selected community sample of multi-ethnic Malay speaking men and women.

The results of our validation study showed that the newly developed CHBMS-CRC-M is a reliable and valid instrument for measuring beliefs regarding CRC screening among Malay-speaking multi-ethnic Malaysians. Cronbach's alpha coefficients for the three subscales ranged from 0.66 to 0.93, which indicated moderate to high internal consistency. The PCA revealed that each item of the scale showed acceptable item correlations (greater than the criterion of 0.30). The relevant items from the 18-item pool loaded separately and sufficiently onto three factors or components in a pattern and manner that was consistent with the three subscales of perceived susceptibility to CRC, perceived benefits of CRC screening and perceived barriers to CRC screening. Barriers tend to vary in nature (eg emotional, practical, service and financial barriers) and a given barrier may have more, less or the same perceived salience for different respondents depending on a range of psychosocial health and life experiences (with resultant implications for intervention design and delivery). Arguably, given this point, it is surprising that a Cronbach's alpha for perceived barriers domain of 0.66 was achieved albeit less than the convention of <0.7. We interpreted 0.6 as indicating an acceptable moderate level of internal reliability (in keeping with other researchers eg Taber, 2018) and viewed the perceived barriers domain to be a single discrete domain that was consistent with the HBM[20].

For peer review only - http://bmjopen.bmj.com/site/about/guidelines.xhtml

For peer review only

The new measure appears to have good structural and construct validity for the assessment of beliefs about CRC and CRC screening as well as good face validity and internal reliability. Our findings are in broad agreement with a study in Korea that translated and validated the CHBMS for colorectal cancer screening[42]. However, the analysis of the much longer 45-item Korean version generated an additional domain or component (in addition to the above three components) that assessed beliefs related to personal self-efficacy. In contrast, our study led to a shorter version of CHBMS that was adapted, translated, and validated to be applicable in the Malay speaking community. We recognise the long-established importance of self-efficacious beliefs to performing positive health behaviours and avoiding harmful behaviours and, so, researchers may wish to avail of the option of adding one of the many existing short, easy to use well-validated self-efficacy scales for cancer screening though there is a need to be mindful of respondent burden and keeping respondents engaged sufficiently to achieve the goal of CRC screening uptake.

The newly developed CHBMS-CRC-M fills an important gap by providing a robust scale with which to investigate and assess CRC screening beliefs and, so, contribute to the development and implementation of culturally sensitive and effective programmes that will enhance CRC screening uptake and early detection of CRC in Malaysia. This scale is suitable, too, for use with other Malay speaking communities in the region such as Indonesia, Singapore, Philippine, southern Thailand, and southern Myanmar.

Strengths and limitations

This study applied a thorough research-informed validation process to develop the new CHBMS-CRC-M scale . The reasonably large, and randomly selected sample from the different ethnic groups was recruited to ascertain cultural acceptability and applicability across the multi-ethnic Malay speaking people in Malaysia.

For peer review only - http://bmjopen.bmj.com/site/about/guidelines.xhtml

For peer review only

Limitation is that, respondents were recruited from Selangor State in Malaysia, and the scale/questionnaire has not been tested with non-Malay speaking ethnic groups. .

Conclusion

There is an urgent need to improve CRC screening uptake in Malaysia and efforts need to be made to address negative beliefs and barriers to CRC screening., The newly developed CHBMS-CRC-M is a reliable and valid scale to measure the beliefs on CRC screening in Malay speaking communities in Malaysia. In addition to using the scale in public health research and service development, the information gathered by using CHBMS-CRC-M may prove to be useful for primary care physicians and staff in terms of helping to identify and reduce misperceptions and barriers to cancer screening, thereby facilitating attendance and improving uptake.

Authors' Contributions:

MDo and TTS conceptualized and planned the project with consultation with VC. TTS, MDa, SYL and DS conducted expert panel discussion and cultural adaptation process. FOA conducted the analysis. FOA, CC, MNNH and TTS interpreted the results. TTS drafted the manuscript with MDo and FOA. All authors reviewed and approved the final manuscript.

Competing interests:

The authors declare no conflict of interest.

Funding:

This research was supported by the UK Medical Research Council—Newton Ungku Omar Funding (MR/P013910/1) and the MRC UKRI GCRF (MR/S014349/1) The funder had no role in the design of the study, collection, analysis, and interpretation of data or writing the manuscript.

Data availability statement:

Data are available from the corresponding author, TT Su, upon reasonable request.

Ethics approval:

For peer review only - http://bmjopen.bmj.com/site/about/guidelines.xhtml

Ethics approval was granted by the Medical Research Ethics Committee, University Malaya Medical Centre (ID: 2016126–4668) and by the National Medical Research Register (ID: NMRR-17-2788-35,613).

Acknowledgement:

The authors would like to acknowledge the support of Dr Nor Saleha Binti Ibrahim Tamin, and Dr Saunthari Somasundaram for the expert panel discussion and Ms Darishiani Paramasivam for systematic review and field data collection.

For peer review only

For peer review only - http://bmjopen.bmj.com/site/about/guidelines.xhtml

References

1. Azizah A, Saleh ITN, Hashima AN, et al. Malaysian National Cancer Registry Report 2007−2011. Malaysia Cancer Statistics, Data and Figure. Putrajaya, Malaysia: National Cancer Institute, Ministry of Health 2016.
2. International Agency for Research on Cancer. GLOBOCAN 2012 v1.0: estimated cancer incidence, mortality and prevalence worldwide in 2012. IARC CancerBase No. 11. globocan.iarc.fr: Cancer Incidence and Mortality Worldwide 2012.
3. International Agency for Research on Cancer. GLOBOCAN 2020. Malaysia Fact Sheets ttps://gco.iarc.fr/today/data/factsheets/populations/458-malaysia-fact-sheets.pdf (accessed 24th February 2022)
4. Zauber AG. The impact of screening on colorectal cancer mortality and incidence: has it really made a difference? *Dig Dis Sci* 2015;60(3):681-91. doi: 10.1007/s10620-015-3600-5 [published Online First: 2015/03/05]
5. Veettil SK, Lim KG, Chaiyakunapruk N, et al. Colorectal cancer in Malaysia: Its burden and implications for a multiethnic country. *Asian journal of surgery* 2017;40(6):481-89.
6. Naing C, Jun YK, Yee WM, et al. Willingness to take a screening test for colorectal cancer: a community-based survey in Malaysia. *European journal of cancer prevention* 2014;23(2):71-75.
7. Suan MAM, Mohammed NS, Abu MR. Colorectal cancer awareness and screening preference: a survey during the Malaysian World Digestive Day Campaign. *Asian Pac J Cancer P* 2015;16(18):8345-49.
8. Hilmi I, Hartono JL, Goh K. Negative perception in those at highest risk--potential challenges in colorectal cancer screening in an urban asian population. *Asian Pac J Cancer Prev* 2010;11(3):815-22.
9. Ho G, Taib N, Singh RP, et al. What if all patients with breast cancer in Malaysia have access to the best available care: how many deaths are avoidable? *Glob J Health Sci* 2017;9(8):32-9.
10. Khan TM, Leong J, Ming LC, et al. Association of knowledge and cultural perceptions of Malaysian women with delay in diagnosis and treatment of breast cancer: a systematic review. *Asian Pac J Cancer Prev* 2015;16(13):5349-57.
11. Yu FQ, Murugiah MK, Khan AH, et al. Meta-synthesis exploring barriers to health seeking behaviour among Malaysian breast cancer patients. *Asian Pac J cancer prev* 2015;16(1):145-52.
12. Azeem E, Gillani SW, Poh V, et al. Barriers to colorectal cancer screening in Asia: a systematic review. *Tropical Journal of Pharmaceutical Research* 2016;15(7):1543-48.
13. Champion VL. Instrument development for health belief model constructs. *Advances in Nursing Science* 1984;6(3):73-85.
14. Champion VL. Instrument refinement for breast cancer screening behaviors. *Nurs Res* 1993;42(3):139-43. doi: 10.1097/00006199-199305000-00003
15. Champion V. Development of a benefits and barriers scale for mammography utilization. *Cancer Nurs* 1995;18(1):53-59.
16. Champion VL, Scott CR. Reliability and validity of breast cancer screening belief scales in African American women. *Nurs Res* 1997;46(6):331-37.
17. Poss JE. Developing a new model for cross-cultural research: synthesizing the health belief model and the theory of reasoned action. *Advances in Nursing Science* 2001;23(4):1-15.
18. Mikhail BI, Petro-Nustas WI. Transcultural adaptation of Champion's health belief model scales. *J Nurs Scholarship* 2001;33(2):159-65.
19. Lee E-H, Kim J-S, Song MS. Translation and validation of Champion's health belief model scale with Korean women. *Cancer Nurs* 2002;25(5):391-95.
20. Champion VL. Revised susceptibility, benefits, and barriers scale for mammography screening. *Res Nurs Health* 1999;22(4):341-48.
21. Champion V, Huster G. Effect of interventions on stage of mammography adoption. *J Behav Med* 1995;18(2):169-87. doi: 10.1007/bf01857868

For peer review only - http://bmjopen.bmj.com/site/about/guidelines.xhtml

22. Vietri V, Poskitt S, Slaninka SC. Enhancing breast cancer screening in the university setting. *Cancer Nurs* 1997;20(5):323-29.
23. Sadler GR, Dong HS, Ko CM, et al. Vietnamese American women: breast cancer knowledge, attitudes, and screening adherence. *American Journal of Health Promotion* 2001;15(4):211-14.
24. Rosenstock IM, Strecher VJ, Becker MH. The health belief model and HIV risk behavior change. In: Peterson J, DiClemente R, eds. Preventing AIDS: Theory and Practice of Behavioural Interventions. New York: Plenum 1994:5-24.
25. Parsa P, Kandiah M, Zulkefli NAM, et al. Knowledge and behavior regarding breast cancer screening among female teachers in Selangor, Malaysia. *Asian Pac J Cancer P* 2008;9(2):221-28.
26. Htay MNN, Donnelly M, Schliemann D, et al. Translation and Validation of the Breast Cancer Awareness Measurement Tool in Malaysia (B-CAM-M). *Asian Pac J Cancer P* 2020;21(1):217-23.
27. Paramasivam D, Schliemann D, Dahlui M, et al. Breast and colorectal cancer awareness in Malaysians and barriers towards screening: A systematic review. medRxiv preprint 2022 doi: https://doi.org/10.1101/2022.02.21.22271312
28. Schliemann D, Donnelly M, Dahlui M, et al. The 'Be Cancer Alert Campaign': protocol to evaluate a mass media campaign to raise awareness about breast and colorectal cancer in Malaysia. *BMC cancer* 2018;18(1):881.
29. Field A. Discovering statistics using IBM SPSS statistics. 4th ed. Los Angeles: Sage Publications 2013.
30. Nunnally JC, Bernstein IH. Psychometric theory. New York: McGraw-Hill Inc 1994.
31. Taber KS. The Use of Cronbach's Alpha When Developing and Reporting Research Instruments in Science Education. Res Sci Educ. 2018; 48, 1273–1296. https://doi.org/10.1007/s11165-016-9602-2
32. Al-Naggar RA, Al-Kubaisy W, Yap BW, et al. Attitudes towards colorectal cancer (CRC) and CRC screening tests among elderly Malay patients. *Asian Pac J Cancer P* 2015;16(2):667-74.
33. Yusoff HM, Daud N, Noor NM, et al. Participation and Barriers to Colorectal Cancer Screening in Malaysia. *Asian Pacific Journal of Cancer Prevention* 2012;13(8):3983-87. doi: 10.7314/apjcp.2012.13.8.3983
34. Tabachnick BG, Fidell LS. Using multivariate statistics. 6 ed. Boston, USA: Pearson 2007.
35. International Business Machines Corporation (IBM). SPSS Statistics, Factor Analysis Extraction. 2016. Accessed on 1 January 2022. Available: https://www.ibm.com/docs/en/spss-statistics/24.0.0?topic=analysis-factor-extraction
36. Allen M. The sage encyclopedia of communication research methods. Thousand Oaks, CA: SAGE Publications. 2017. doi: 10.4135/9781483381411
37. Htay MNN, Schliemann D, Dahlui M, et al. Validation of the Champion Health Belief Model Scale for an Investigation of Breast Cancer Screening Behaviour in Malaysia. *Int J Environ Res Public Health*. 2021;18(17):9311.
38. Ministry of Health Malaysia P. Management of Colorectal Carcinoma: Putrajaya, 2017.
39. Van Tze CN, Fitzgerald H, Qureshi A, et al. Understanding colorectal cancer in Malaysia: A mini-review and pioneering colorectal cancer awareness, screening and treatment project. 2017
40. Magaji BA, Moy FM, Roslani AC, et al. Survival rates and predictors of survival among colorectal cancer patients in a Malaysian tertiary hospital. *BMC cancer* 2017;17(1):339.
41. Lau J, Lim TZ, Wong GJ, Tan KK. The health belief model and colorectal cancer screening in the general population: A systematic review. *Prev Med Rep.* 2020; 20:101223. doi: 10.1016/j.pmedr.2020.101223
42. Lee SY, Lee EE, Rhee YS. Adaptation and validation of the health belief model scale for colorectal cancer screening. *Nurs Health Sci.* 2020;22:355–363.

For peer review only

BMJ Open

Testing the validity of a new scale designed to assess beliefs and perceptions about colorectal cancer and colorectal cancer screening in Malaysia: A principal component analysis

Journal:	*BMJ Open*
Manuscript ID	bmjopen-2023-072166.R1
Article Type:	Original research
Date Submitted by the Author:	24-May-2023

Complete List of Authors:	Su, Tin ; Monash University - Malaysia Campus, Jeffrey Cheah School of Medicine and Health Sciences<br>Adekunjo, Felix ; Lagos State University, Department of Economics, Faculty of Social Sciences<br>Schliemann, Desiree ; Queen's University Belfast, Centre for Public Health and UKCRC Centre of Excellence for Public Health<br>Cardwell, Christopher; Queen's University Belfast, Centre for Public Health and UKCRC Centre of Excellence for Public Health<br>Htay , Mila; University of Malaya, Department of Social and Preventive Medicine, Faculty of Medicine<br>Dahlui, Maznah; Universiti Malaya, Department of Social and Preventive Medicine, Faculty of Medicine<br>Loh, Siew_Yim; University of Malaya, Department of Rehabilitation Medicine, Faculty of Medicine<br>Champion, Victoria; Indiana University, School of Nursing<br>Donnelly, Michael; Queen's University Belfast, Centre for Public Health and UKCRC Centre of Excellence for Public Health

**Primary Subject Heading**:	Public health
Secondary Subject Heading:	Epidemiology, Oncology, Research methods
Keywords:	PUBLIC HEALTH, EPIDEMIOLOGY, Cancer pain < ONCOLOGY

SCHOLARONE™
Manuscripts

I, the Submitting Author has the right to grant and does grant on behalf of all authors of the Work (as defined in the below author licence), an exclusive licence and/or a non-exclusive licence for contributions from authors who are: i) UK Crown employees; ii) where BMJ has agreed a CC-BY licence shall apply, and/or iii) in accordance with the terms applicable for US Federal Government officers or employees acting as part of their official duties; on a worldwide, perpetual, irrevocable, royalty-free basis to BMJ Publishing Group Ltd ("BMJ") its licensees and where the relevant Journal is co-owned by BMJ to the co-owners of the Journal, to publish the Work in this journal and any other BMJ products and to exploit all rights, as set out in our licence.

The Submitting Author accepts and understands that any supply made under these terms is made by BMJ to the Submitting Author unless you are acting as an employee on behalf of your employer or a postgraduate student of an affiliated institution which is paying any applicable article publishing charge ("APC") for Open Access articles. Where the Submitting Author wishes to make the Work available on an Open Access basis (and intends to pay the relevant APC), the terms of reuse of such Open Access shall be governed by a Creative Commons licence – details of these licences and which Creative Commons licence will apply to this Work are set out in our licence referred to above.

Other than as permitted in any relevant BMJ Author's Self Archiving Policies, I confirm this Work has not been accepted for publication elsewhere, is not being considered for publication elsewhere and does not duplicate material already published. I confirm all authors consent to publication of this Work and authorise the granting of this licence.

For peer review only

Testing the validity of a new scale designed to assess beliefs and perceptions about colorectal cancer and colorectal cancer screening in Malaysia: a principal component analysis

Tin Tin Su[1,2, 4*], Felix Oluyemi Adekunjo[3], Désirée Schliemann[4], Christopher Cardwell[4], Mila Nu Nu Htay[2,5], Maznah Dahlui[2], Siew Yim Loh[6] , Victoria Champion[7] and Michael Donnelly[4*]

Affiliation:

1) South East Asia Community Observatory (SEACO) & Global Public Health, Jeffery Cheah School of Medicine and Health Sciences, Monash University Malaysia, Bandar Sunway, Malaysia

2) Centre for Population Health (CePH), Department of Social and Preventive Medicine, Faculty of Medicine, University of Malaya, Kuala Lumpur, Malaysia

3) Department of Economics, Faculty of Social Sciences, Lagos State University, Lagos, Nigeria

4) Centre for Public Health and UKCRC Centre of Excellence for Public Health, Queen's University Belfast, UK

5) Department of Community Medicine, Faculty of Medicine, Manipal University College Malaysia, Jalan Batu Hampar, 75150 Bukit Baru, Melaka, Malaysia

6) Department of Rehabilitation Medicine, Faculty of Medicine, University of Malaya, Kuala Lumpur, Malaysia

7) Indiana University, School of Nursing, USA

* Michael Donnelly (Corresponding author)

Centre for Public Health and UKCRC Centre of Excellence for Public Health, Queen's University Belfast, UK

Phone: +44 (0)28 9097 8993

Michael.Donnelly@qub.ac.uk

*Tin Tin Su (Corresponding author)

For peer review only - http://bmjopen.bmj.com/site/about/guidelines.xhtml

Affiliation: South East Asia Community Observatory (SEACO) & Global Public Health, Jeffery Cheah School of Medicine and Health Sciences, Monash University Malaysia, Bandar Sunway, Malaysia

Department of Social and Preventive Medicine, University of Malaya, Kuala Lumpur, Malaysia

Centre for Public Health and UKCRC Centre of Excellence for Public Health, Queen's University Belfast, UK

Phone: +603-5514 447

*** TinTin.Su@monash.edu**

Running title: A questionnaire to assess beliefs about colorectal cancer and screening

For peer review only

For peer review only - http://bmjopen.bmj.com/site/about/guidelines.xhtml

Abstract

Objective: To conduct a cultural adaptation and validation of the Champion Health Belief Model Scale (CHBMS) for colorectal (CRC) cancer screening (CHBMS-CRC-M) in order to assess and investigate perceptions and beliefs about CRC screening in Malaysia.

Designs and participants: The results from an evidence synthesis and the outcomes from an expert panel discussion were used to shape CHBMS scale content into an assessment of beliefs about CRC screening (CHBMS-CRC). This questionnaire assessment was translated into the official language of Malaysia. An initial study tested the face validity of the new scale or questionnaire with 30 men and women from various ethnic groups. Factorial or structural validity was investigated in a community sample of 954 multi-ethnic Malaysians.

Setting: Selangor state, Malaysia

Results: The new scale was culturally acceptable to the three main ethnic groups in Malaysia and achieved good face validity. Cronbach's alpha coefficients ranged from 0.66 to 0.93, indicating moderate to good internal consistency. Items relating to perceived susceptibility (PSU) to CRC 'loaded' on Factor 1 (with loadings scoring above 0.90); perceived benefits (PBE) of CRC screening items loaded on Factor 2 and were correlated strongly (loadings ranged between 0.63 and 0.83); and perceived barriers (PBA) to CRC screening (PBA) items loaded on Factor 3 (range: 0.30 to 0.72).

Conclusion: The newly developed CHBMS-CRC-M fills an important gap by providing a robust scale with which to investigate and assess CRC screening beliefs and contribute to efforts to enhance CRC screening uptake and early detection of CRC in Malaysia and in other Malay speaking communities in the region.

Keywords: Beliefs, colorectal cancer, cancer screening, questionnaire validation, cultural adaptation, Malaysia

For peer review only

Strengths and Limitations

- The development of the new CHBMS-CRC-M scale was informed by an evidence synthesis and iterative review by a multidisciplinary expert panel
- We followed the procedures set out in the relevant WHO guidelines for the Process of translation and adaptation of instruments and the Cross-Cultural Survey Guidelines
- A large randomly selected sample from different ethnic groups was recruited
- Respondents were recruited from one state in Malaysia
- The questionnaire has not been tested for use with non-Malay-speaking ethnic groups

For peer review only

For peer review only - http://bmjopen.bmj.com/site/about/guidelines.xhtml

Introduction

The global burden of colorectal cancer (CRC) is increasing. Appropriate cancer control measures are needed to reduce increasing CRC mortality particularly in LMICs such as Malaysia where CRC is the second most common cancer[1]. According to GLOBOCAN report 2020, 6,597 CRC cases were detected in 2020 (13.6% of all newly diagnosed cancer cases) and CRC-related deaths rose from 2,565 to 3,420 within 8 years. [2,3]. It is estimated that about one-third to one-half of deaths due to CRC could be avoided through early presentation, detection and appropriate treatment[4]. However, over 50% of CRC patients in Malaysia present at late stages[5]. Research indicates that only 8% of people aged ≥50 years attend CRC screening, understanding about CRC screening tests is lacking[6 7] and willingness to participate in CRC screening is low[8]. According to Ho, et al. [9], about half of cancer-related deaths in Malaysia could be avoided if cancer was detected and diagnosed early. Studies indicate that negative beliefs and perceptions may play a role in late presentation and diagnosis of advanced cancer among Malaysians[10-12]. Thus, there is a need to develop a reliable and valid measurement tool to investigate perceptions and beliefs about screening.

The Health Belief Model (HBM) provided the theoretical framework for this study[7]. Previous studies applied the model to breast screening (BC) behaviour and, subsequently, the Champion Health Belief Model Scale (CHBMS-BC) was developed[13]. Further refinements and revisions led initially to the addition of a subscale - confidence regarding breast self-examination[14] and the addition of item questions to capture mammogram screening. The final revised CHBMS-BC for mammogram screening included three subscales: perceived susceptibility, benefits and barriers[15]. The CHBMS-BC has been translated into different languages and adapted and tested in different countries[16-19] including being used to assess the effectiveness of interventions which targeted women's participation in BC screening programme[20-24]. In addition, the

For peer review only - http://bmjopen.bmj.com/site/about/guidelines.xhtml

CHBMS-BC has been adapted and validated to assess perceptions about BC screening among Malaysian women[25 26].

As far as we are aware, a measure of the beliefs about colorectal cancer screening in Malaysia does not exist. The purpose of this study was to conduct a cultural adaptation of the CHBMS-BC questionnaire to develop a validated tool to investigate CRC screening beliefs in Malaysia

Method

Evidence synthesis

We conducted a systematic review (according to PRISMA guidelines and a protocol was registered with PROSPERO) in order to synthesize the findings from Malaysia-based studies that investigated CRC symptoms awareness and barriers to cancer screening uptake[27].

Expert panel discussion

The CHBMS for mammogram screening was adapted for use in relation to CRC screening. An expert panel conducted the adaptation process based on the findings of the systemic review, differences in the nature of screenings and consideration of local scenarios. The panel of experts included three public health physicians, two cancer epidemiologists, one cancer advocate and one psychologist.

Linguistic validation

The translation of the adapted scale (CHBMS-CRC-M) followed the procedures set out in the relevant WHO guidelines (*WHO | Process of translation and adaptation of instruments*, n.d.) and the Cross-Cultural Survey Guidelines (CCSG) (*Adaptation—Cross-Cultural Survey Guidelines*, n.d.). The questionnaire was translated into the local and official language of Malaysia (Bahasa Melayu). Two independent professional translators who are bilingual (English and Bahasa Melayu) conducted forward translation. We worked towards achieving a conceptual and culturally appropriate version rather than a literal translation. One of the

For peer review only

researchers, who is a native Malay speaker, compared both versions and resolved any discrepancies via discussions with the translators.

Initial testing phase

We tested the face validity of the questionnaire with 30 (male/female) participants from the major ethnic groups in Malaysia (Malay, Chinese & Indian) in order to evaluate the extent to which the questionnaire was clear, simple and easy to understand, the presence of any inappropriate, redundant, incorrectly worded or missing items and the relevancy, flow and arrangement of the questionnaire. Trained interviewers undertook face-to-face interviews. Generally, the face validity study indicated that the newly developed CHBMS-CRC-M questionnaire was culturally acceptable to ethnic Malaysian men and women.

Main study phase

The culturally validated CHBMS-CRC-M questionnaire was administered during the baseline data collection phase of the *Be Cancer Alert Campaign* (BCAC) [28]. The baseline survey occurred between January and March 2018 in Rawang sub-district of the Gombak, Selangor State - the most populated state in Malaysia with a population above 5 million. Rawang sub-district was chosen (in consultation with the Department of Statistics: DOSM) because it contains a representative mix of ethnicities, age groups and income groups. The target sample comprised adult males and females who 1) lived in randomly selected households, 2) were above 40 years of age, 3) were fluent in Malay and 4) were able to answer independently without support from others. The age-range was selected because populations in low-middle-income countries (LMIC) including Malaysia tend to be diagnosed with cancer at a younger age compared to high income countries. The DOSM randomly selected 4,368 houses from the 273 Enumeration Blocks (EBs - artificially created contiguous geographical areas with specific boundaries containing 100 households). We oversampled to take account of non-resident areas, business premises, non-respondents and survey-ineligible residents. The

For peer review only - http://bmjopen.bmj.com/site/about/guidelines.xhtml

security guards and management offices in certain housing areas did not allow the survey to be conducted (this comprised a total of 656 houses across 36 EBs); 65 houses were non-residential business premises; and 253 houses were unoccupied. The survey was conducted during official day time working hours due to our duty to ensure the safety of our interviewers. There did not appear to be residents at home in 1,635 houses, most likely, because residents were working adults and were at their place of work when our interviewers called to these homes. A total of 1,292 out of 1,759 reachable households agreed to participate - a response rate of 73%. Finally, 954 participants who fulfilled the above-mentioned inclusion criteria completed the survey and the new CHBMS-CRC-M questionnaire. The percentage of missing data relating to items ranged from (0.4 – 2.7 %).

Trained and supervised research assistants conducted face-to-face interviews after obtaining written informed consent from participants.

Data analysis

SPSS v22.0 statistical software was used to conduct descriptive statistics and a factor analysis (using a principle component analysis, varimax rotation, an eigenvalue of 1 and above, and 0.3 as a cut-off point for loading onto a factor). Sampling adequacy was assessed using Kaiser-Meyer-Olkin (KMO), Bartlett's test of sphericity and a KMO value of 0.70 and above [29 30]. Reliability was assessed using Cronbach's Alpha as a test of the internal consistency of items under each component of the scale: perceived susceptibility to CRC screening (PSU), perceived benefits of CRC screening (PBE) and perceived barriers to CRC screening (PBA). We interpreted Cronbach Alpha scores to indicate as follows: 0.61-0.65 - moderate, 0.71-0.91 - good, and 0.91-0.93 - strong internal consistency in keeping with Taber (2018) [31].

Patient and Public Involvement

For peer review only - http://bmjopen.bmj.com/site/about/guidelines.xhtml

For peer review only

Key stakeholders and researchers with experience in cancer prevention and control were consulted at various stages including the cultural adaptation phase and during face validity testing of the new scale. The newly developed CHBMS-CRC-M will be posted on the research team's website (http://www.becanceralert.com/research/bcac/) and will be available freely for use by researchers and practitioners.

Results

Evidence synthesis

Nine studies met review eligibility criteria but only three studies included data about barriers to CRC screening uptake. Each one of the three studies used a different self-developed questionnaire to assess barriers to screening and one study included data about domains related to the HBM in terms of perceived susceptibility and benefits of screening[6 32 33]. "Fear of result" from colorectal screening was the most commonly reported barrier in the three studies. Two studies reported the following comments in common which potentially could act as barriers or inhibiting factors: "screening is embarrassing" (35% and 55% respectively), "did not have symptoms of CRC" (13% and 39% respectively) and "FOBT was not recommended by doctor" (11% and 35% respectively)[30 31]. Similarly, a further two studies from the review documented that "fear of discomfort" during CRC screening (30% and 64% respectively) and "CRC screening is expensive" (23% and 53% respectively) [6 33].

Expert panel discussion

The expert panel used the three subscales (susceptibility, benefits, and barriers) of the original scale to produce the 18-item CHBMS-CRC-M. A comparison of the item content of the original CHBMS and the newly adapted and developed CHBMS-CRC-M is presented in Table 1. For example, the perceived barriers subscale to colorectal cancer screening comprised 11 items which covered emotional, practical, service and financial barriers. The response format to every item was a 5-point Likert Scale: (1) represented 'strongly disagree' while (5) represented strongly agree. Scores on each domain or subscale ranged as follows: susceptibility (3-15),

For peer review only - http://bmjopen.bmj.com/site/about/guidelines.xhtml

benefits (4-20) and barriers (11-55). The explanatory note about CRC screening read: *"Colorectal cancer screening is carried out to test whether or not someone has cancer. Colorectal cancer can be detected through an examination of a stool and/or by examining a patient's colon and rectum with a camera that is inserted through their posterior or backside."*

For peer review only

For peer review only - http://bmjopen.bmj.com/site/about/guidelines.xhtml

Table.1 Comparison of items from the original CHBMS for mammogram screening and newly developed CHBMS-CRC-M

Original CHBMS for mammogram screening	CHBMS_CRC-M
Susceptibility	
1. It is likely that I will get breast cancer.	1. It is likely that I will get colorectal cancer.
2. My chances of getting breast cancer in the next few years are great.	2. My chances of getting colorectal cancer in the next few years are high.
3. I feel I will get breast cancer sometime during my life.	3. I feel I will get colorectal cancer sometime during my life.
Benefits	
1. If I get a mammogram and nothing is found, I do not worry as much about breast cancer.	1. If I get colorectal cancer screening and nothing is found, I will worry less about colorectal cancer.
2. Having a mammogram will help me find breast lumps early.	2. Having colorectal cancer screening will help me find colorectal cancer early.
3. If I find a lump through a mammogram, my treatment for breast cancer may not be as bad.	3. If I find abnormalities through colorectal cancer screening my treatment for colorectal cancer may not be as bad.
4. Having a mammogram is the best way for me to find a very small lump.	
5. Having a mammogram will decrease my chances of dying from breast cancer.	4. Having colorectal cancer screening will decrease my chances of dying from colorectal cancer.
Barriers	
1. I am afraid to have a mammogram because I might find out something is wrong.	1. I am afraid to have colorectal cancer screening because I might find out something is wrong.
2. I am afraid to have a mammogram because I don't understand what will be done.	2. I am afraid to have colorectal cancer screening because I don't understand what will be done.
3. I don't know how to go about getting a mammogram.	3. I don't know how to go about getting colorectal cancer screening.
4. Having a mammogram is too embarrassing.	4. Having colorectal cancer screening is too embarrassing.
5. Having a mammogram takes too much time.	5. Having colorectal cancer screening takes too much time.
6. Having a mammogram is too painful.	6. Having colorectal cancer screening is too painful.
7. People doing mammograms are rude to women.	7. People doing colorectal cancer screening are rude to patients.
8. Having a mammogram exposes me to unnecessary radiation.	
9. I cannot remember to schedule a mammogram.	
10. I have other problems more important than getting a mammogram.	8. I have other problems more important than getting colorectal cancer screening.
11. I am too old to need a routine mammogram.	9. I am not the right age to need a routine colorectal cancer screening.
	10. I cannot afford to get colorectal cancer screening.
	11. I don't have the encouragement I need from my close relatives to attend colorectal cancer screening.

For peer review only - http://bmjopen.bmj.com/site/about/guidelines.xhtml

The final data analysis comprised a sample of 954 survey participants. Table 2 presents descriptive statistics for the sample. For example, 62% were male, 82% were married, around a quarter had at least above certificate education level; mean age was 50.84 years (SD = 16.85) and, on average, there were five residents per household (SD = 2.28).

Table 2: Sociodemographic characteristics (n=954)

Characteristic	Number	Percentage
**Gender**		
Male	361	38
Female	593	62
**Nationality**		
Malaysian	871	91
Non-Malaysian	80	8
**Marital Status**		
Single	31	3
Married	783	82
Divorcee & Widow	136	15
**Religion**		
Islam	585	61
Christianity	35	4
Buddhism	95	10
Hinduism	226	24
Others	11	1.1
**Ethnicity**		
Malay	516	54
Chinese	110	12
Indian	264	28
Others	64	6.7
**Education**		
No formal education	73	8
Never completed primary school	79	8
Completed primary school	190	20
Completed secondary school	386	41
Certificate A-Level/STPM/HSC	99	10
Tertiary education	125	13
**Current Job**		
Civil servant	26	3
Private sector employee	194	20
Self-employed	128	13
Retiree	116	12
Homemakers	377	40
Unemployed	113	12
**Main Occupation**		
Manager	13	1
Professionals	50	5
Technicians and associate professionals	31	3
Clerical support workers	20	2
Service and sales workers	110	12
Skilled agricultural, forestry and fishery workers	6	.6
Craft and related trades workers	5	.5
Plant and machine-operators and assemblers	19	2

For peer review only

For peer review only - http://bmjopen.bmj.com/site/about/guidelines.xhtml

Elementary occupations<br>Healthcare professional (i.e. doctors, nurses, etc)	87<br>5	9<br>.5
**Monthly Family Income**<br>Below RM 2, 000<br>RM 2, 000 - RM 3, 000<br>RM 3, 000 - RM 4, 000<br>Above RM 4, 000	 342<br>201<br>118<br>147	 36<br>21<br>12<br>16

Principal Component Analysis

The sample size (n=954) in relation to the new 18-item scale and 5-point Likert response was more than required in order to conduct a robust principal component analysis (PCA)[34]. A PCA was conducted on the 18 items of the CHBMS with varimax rotation. We applied a PCA to obtain the component or factor solution based on the linear combination of observed items [35].

Varimax rotation was used to clarify the relationships between components and items[36]. Researchers applied PCA and varimax rotation during the validation of the original CHBMS for breast cancer screening [20], and the translated Malay version of CHBMS-BC-M [37]. We followed a similar analytic process to the process that was used in the previously published articles (referenced above) in keeping with the original theoretically-informed investigative approach to BC and mammogram screening. A key aim of the analysis was to test the appropriateness and acceptability of the adapted and supplemented item content in the context of a very different type of cancer from BC; and to conduct a PCA to investigate the nature and degree to which the item content grouped together to represent or explain beliefs about CRC and potential use of CRC screening. Ideally, further field testing would occur and at some later stage a confirmatory factor analysis might be undertaken.

The Kaiser-Meyer-Olkin test verified sampling adequacy for the conduct of a PCA (KMO= 0.73) and Bartlett's test of sphericity (Chi-square = 5093.73) confirmed that the sample size

For peer review only - http://bmjopen.bmj.com/site/about/guidelines.xhtml

was adequate. The analysis obtained eigenvalues for the factors each of which exceeded Kaiser's criterion of 1 and in combination explained 44% of variance (Table 3).

Table 3: Total Variance Explained

Item	Initial Eigenvalues			Extraction Sums of Squared Loadings			Rotation Sums of Squared Loadings		
	Total	% of Variance	Cumulative %	Total	% of Variance	Cumulative %	Total	% of Variance	Cumulative %
1	2.966	16.476	16.476	2.966	16.476	16.476	2.685	14.914	14.914
2	2.682	14.898	31.374	2.682	14.898	31.374	2.672	14.842	29.756
3	2.182	12.123	43.497	2.182	12.123	43.497	2.473	13.740	43.497
4	1.393	7.738	51.234						
5	1.089	6.048	57.282						
6	1.046	5.810	63.092						
7	.923	5.130	68.222						
8	.874	4.856	73.078						
9	.777	4.317	77.395						
10	.731	4.059	81.453						
11	.643	3.573	85.027						
12	.632	3.514	88.540						
13	.569	3.159	91.700						
14	.474	2.631	94.331						
15	.380	2.112	96.443						
16	.278	1.547	97.990						
17	.250	1.388	99.379						
18	.112	.621	100.000						

For peer review only - http://bmjopen.bmj.com/site/about/guidelines.xhtml

Items relating to perceived susceptibility to CRC (PSU) 'loaded' on Factor 1 (with loadings above 0.90); perceived benefits of CRC screening (PBE) items loaded on Factor 2 and were correlated strongly (loadings ranged between 0.63 and 0.83); and perceived barriers to CRC screening (PBA) items loaded on Factor 3 (range: 0.30 to 0.72). Table 4 presents the rotated factor matrix and three-factor solution (items greater than 0.3 were retained as recommended by best practice) [30].

Table 4. CHBMS-CRC-M item loadings for each factor

Subscales and items	Factor loadings	Factor loadings	Factor loadings
Susceptibility			
PSU1	.944		
PSU2	.943		
PSU3	.903		
Benefits			
PBE1		.627	
PBE2		.826	
PBE3		.799	
PBE4		.760	
Barriers			
PBA1			.716
PBA2			.702
PBA3			.305
PBA4			.619
PBA5			.391
PBA6			.429
PBA7			.428
PBA8			.533
PBA9			.419
PBA10			.304
PBA11			.321

Internal reliability or consistency

The perceived susceptibility to CRC (PSU) indicated high reliability (Cronbach's α = 0.93, with all items strongly correlated around 0.80); the perceived benefits of CRC screening (PBE)

For peer review only - http://bmjopen.bmj.com/site/about/guidelines.xhtml

had fairly high overall internal consistency (Cronbach's α = 0.78 though inter-item correlations ranged from 0.31 to 0.57); and the reliability test for perceived barriers to CRC screening was moderate (Cronbach's α = 0.66 with some inter-item correlations below 0.30).

Discussion

Although annual CRC screening is recommended for people aged ≥50 years in Malaysia [38], a population-based screening programme is not in place and efforts so far to improve uptake have been selective and sporadic [39]. The absence of a population-based screening programme and the development of advanced cancer impacts negatively on survival [5 40], requires more intensive treatment, increases health care resource utilization and places additional financial burden on households. The health care system and context in Malaysia requires systematic research attention in order to ensure the successful implementation of CRC screening, particularly in terms of the promotion, acceptance of, and particpation in, CRC screening among the general population. Misperception of risk of cancer, denial of the existence of the disease, emotional barriers and negative beliefs about cancer screening generally affect screening uptake and lead to delay in seeking medical help.

An in-depth understanding about how adults perceive their susceptibility to CRC, the benefits of attending screening and barriers to CRC screening is crucial for the successful implementation of CRC screening and enhancement of screening coverage. A systematic review comprising 30 articles evaluated the association between HBM domains and CRC screening in the community[41]. The review found that higher scores for the domains, perceived susceptibility and benefits, and lower scores for the barriers domain were associated with positive screening behaviour; and cues to action were associated with CRC screening adherence[41]. The results of the review provide support for the potential benefits of using the

For peer review only - http://bmjopen.bmj.com/site/about/guidelines.xhtml

new scale in terms of improved understanding and providing insights into the design of screening uptake interventions.

We adapted, translated, and tested the CHBMS-CRC-M in order to create a means of evaluating Malaysians' beliefs or perceptions about CRC screening. The cultural adaptation of the CHBMS-CRC-M was grounded robustly via an evidence synthesis, an expert panel discussion and linguistic validation exercise. The scale was proven to have good face validity in a relatively small separate survey from the main study which assessed its structural validity in a large randomly selected community sample of multi-ethnic Malay speaking men and women.

The results of our validation study showed that the newly developed CHBMS-CRC-M is a reliable and valid instrument for measuring beliefs regarding CRC screening among Malay-speaking multi-ethnic Malaysians. Cronbach's alpha coefficients for the three subscales ranged from 0.66 to 0.93, which indicated moderate to high internal consistency. The PCA revealed that each item of the scale showed acceptable item correlations (greater than the criterion of 0.30). The relevant items from the 18-item pool loaded separately and sufficiently onto three factors or components in a pattern and manner that was consistent with the three subscales of perceived susceptibility to CRC, perceived benefits of CRC screening and perceived barriers to CRC screening. Barriers tend to vary in nature (eg emotional, practical, service and financial barriers) and a given barrier may have more, less or the same perceived salience for different respondents depending on a range of psychosocial health and life experiences (with resultant implications for intervention design and delivery). Arguably, given this point, it is surprising that a Cronbach's alpha for perceived barriers domain of 0.66 was achieved albeit less than the convention of <0.7. We interpreted 0.6 as indicating an acceptable moderate level of internal reliability (in keeping with other researchers eg Taber, 2018) and viewed the perceived barriers domain to be a single discrete domain that was consistent with the HBM[20].

For peer review only - http://bmjopen.bmj.com/site/about/guidelines.xhtml

The new measure appears to have good structural and construct validity for the assessment of beliefs about CRC and CRC screening as well as good face validity and internal reliability. Our findings are in broad agreement with a study in Korea that translated and validated the CHBMS for colorectal cancer screening[42]. However, the analysis of the much longer 45-item Korean version generated an additional domain or component (in addition to the above three components) that assessed beliefs related to personal self-efficacy. In contrast, our study led to a shorter version of CHBMS that was adapted, translated, and validated to be applicable in the Malay speaking community. We recognise the long-established importance of self-efficacious beliefs to performing positive health behaviours and avoiding harmful behaviours and, so, researchers may wish to avail of the option of adding one of the many existing short, easy to use well-validated self-efficacy scales for cancer screening though there is a need to be mindful of respondent burden and keeping respondents engaged sufficiently to achieve the goal of CRC screening uptake.

The newly developed CHBMS-CRC-M fills an important gap by providing a robust scale with which to investigate and assess CRC screening beliefs and, so, contribute to the development and implementation of culturally sensitive and effective programmes that will enhance CRC screening uptake and early detection of CRC in Malaysia. This scale is suitable, too, for use with other Malay speaking communities in the region such as Indonesia, Singapore, Philippine, southern Thailand, and southern Myanmar.

Strengths and limitations

This study applied a thorough research-informed validation process to develop the new CHBMS-CRC-M scale. The reasonably large, and randomly selected sample from the different ethnic groups was recruited to ascertain cultural acceptability and applicability across the multi-ethnic Malay speaking people in Malaysia. However, it is important to note that respondents were recruited from Selangor State in Malaysia, and the scale/questionnaire has not been tested with non-Malay speaking ethnic groups. .

For peer review only - http://bmjopen.bmj.com/site/about/guidelines.xhtml
For peer review only

Conclusion

There is an urgent need to improve CRC screening uptake in Malaysia and efforts are needed to address negative beliefs and barriers to CRC screening., The newly developed CHBMS-CRC-M is a reliable and valid scale to measure the beliefs on CRC screening in Malay speaking communities in Malaysia. In addition to using the scale in public health research and service development, the information gathered by using CHBMS-CRC-M may prove to be useful for primary care physicians and staff in terms of helping to identify and reduce misperceptions and barriers to cancer screening, thereby facilitating attendance and improving uptake.

Authors' Contributions:

MDo and TTS conceptualized and planned the project with consultation with VC. TTS, MDa, SYL and DS conducted expert panel discussion and cultural adaptation process. FOA conducted the analysis. FOA, CC, MNNH and TTS interpreted the results. TTS drafted the manuscript with MDo and FOA. All authors reviewed and approved the final manuscript.

Competing interests:

The authors declare no conflict of interest.

Funding:

This research was supported by the UK Medical Research Council—Newton Ungku Omar Funding (MR/P013910/1) and the MRC UKRI GCRF (MR/S014349/1) The funder had no role in the design of the study, collection, analysis, and interpretation of data or writing the manuscript.

Data availability statement:

Data are available from the corresponding authors, M Donnelly and TT Su, upon reasonable request.

Ethics approval:

For peer review only - http://bmjopen.bmj.com/site/about/guidelines.xhtml
For peer review only

Ethics approval was granted by the Medical Research Ethics Committee, University Malaya Medical Centre (ID: 2016126–4668) and by the National Medical Research Register (ID: NMRR-17-2788-35,613).

Acknowledgement:

The authors would like to acknowledge the support of Dr Nor Saleha Binti Ibrahim Tamin, and Dr Saunthari Somasundaram for the expert panel discussion and Ms Darishiani Paramasivam for systematic review and field data collection.

For peer review only

References

1. Azizah A, Saleh ITN, Hashima AN, et al. Malaysian National Cancer Registry Report 2007−2011. Malaysia Cancer Statistics, Data and Figure. Putrajaya, Malaysia: National Cancer Institute, Ministry of Health 2016.
2. International Agency for Research on Cancer. GLOBOCAN 2012 v1.0: estimated cancer incidence, mortality and prevalence worldwide in 2012. IARC CancerBase No. 11. globocan.iarc.fr: Cancer Incidence and Mortality Worldwide 2012.
3. International Agency for Research on Cancer. GLOBOCAN 2020. Malaysia Fact Sheets ttps://gco.iarc.fr/today/data/factsheets/populations/458-malaysia-fact-sheets.pdf (accessed 24th February 2022)
4. Zauber AG. The impact of screening on colorectal cancer mortality and incidence: has it really made a difference? *Dig Dis Sci* 2015;60(3):681-91. doi: 10.1007/s10620-015-3600-5 [published Online First: 2015/03/05]
5. Veettil SK, Lim KG, Chaiyakunapruk N, et al. Colorectal cancer in Malaysia: Its burden and implications for a multiethnic country. *Asian journal of surgery* 2017;40(6):481-89.
6. Naing C, Jun YK, Yee WM, et al. Willingness to take a screening test for colorectal cancer: a community-based survey in Malaysia. *European journal of cancer prevention* 2014;23(2):71-75.
7. Suan MAM, Mohammed NS, Abu MR. Colorectal cancer awareness and screening preference: a survey during the Malaysian World Digestive Day Campaign. *Asian Pac J Cancer P* 2015;16(18):8345-49.
8. Hilmi I, Hartono JL, Goh K. Negative perception in those at highest risk--potential challenges in colorectal cancer screening in an urban asian population. *Asian Pac J Cancer Prev* 2010;11(3):815-22.
9. Ho G, Taib N, Singh RP, et al. What if all patients with breast cancer in Malaysia have access to the best available care: how many deaths are avoidable? *Glob J Health Sci* 2017;9(8):32-9.
10. Khan TM, Leong J, Ming LC, et al. Association of knowledge and cultural perceptions of Malaysian women with delay in diagnosis and treatment of breast cancer: a systematic review. *Asian Pac J Cancer Prev* 2015;16(13):5349-57.
11. Yu FQ, Murugiah MK, Khan AH, et al. Meta-synthesis exploring barriers to health seeking behaviour among Malaysian breast cancer patients. *Asian Pac J cancer prev* 2015;16(1):145-52.
12. Azeem E, Gillani SW, Poh V, et al. Barriers to colorectal cancer screening in Asia: a systematic review. *Tropical Journal of Pharmaceutical Research* 2016;15(7):1543-48.
13. Champion VL. Instrument development for health belief model constructs. *Advances in Nursing Science* 1984;6(3):73-85.
14. Champion VL. Instrument refinement for breast cancer screening behaviors. *Nurs Res* 1993;42(3):139-43. doi: 10.1097/00006199-199305000-00003
15. Champion V. Development of a benefits and barriers scale for mammography utilization. *Cancer Nurs* 1995;18(1):53-59.
16. Champion VL, Scott CR. Reliability and validity of breast cancer screening belief scales in African American women. *Nurs Res* 1997;46(6):331-37.
17. Poss JE. Developing a new model for cross-cultural research: synthesizing the health belief model and the theory of reasoned action. *Advances in Nursing Science* 2001;23(4):1-15.
18. Mikhail BI, Petro-Nustas WI. Transcultural adaptation of Champion's health belief model scales. *J Nurs Scholarship* 2001;33(2):159-65.
19. Lee E-H, Kim J-S, Song MS. Translation and validation of Champion's health belief model scale with Korean women. *Cancer Nurs* 2002;25(5):391-95.
20. Champion VL. Revised susceptibility, benefits, and barriers scale for mammography screening. *Res Nurs Health* 1999;22(4):341-48.
21. Champion V, Huster G. Effect of interventions on stage of mammography adoption. *J Behav Med* 1995;18(2):169-87. doi: 10.1007/bf01857868

For peer review only

22. Vietri V, Poskitt S, Slaninka SC. Enhancing breast cancer screening in the university setting. *Cancer Nurs* 1997;20(5):323-29.
23. Sadler GR, Dong HS, Ko CM, et al. Vietnamese American women: breast cancer knowledge, attitudes, and screening adherence. *American Journal of Health Promotion* 2001;15(4):211-14.
24. Rosenstock IM, Strecher VJ, Becker MH. The health belief model and HIV risk behavior change. In: Peterson J, DiClemente R, eds. Preventing AIDS: Theory and Practice of Behavioural Interventions. New York: Plenum 1994:5-24.
25. Parsa P, Kandiah M, Zulkefli NAM, et al. Knowledge and behavior regarding breast cancer screening among female teachers in Selangor, Malaysia. *Asian Pac J Cancer P* 2008;9(2):221-28.
26. Htay MNN, Donnelly M, Schliemann D, et al. Translation and Validation of the Breast Cancer Awareness Measurement Tool in Malaysia (B-CAM-M). *Asian Pac J Cancer P* 2020;21(1):217-23.
27. Paramasivam D, Schliemann D, Dahlui M, et al. Breast and colorectal cancer awareness in Malaysians and barriers towards screening: A systematic review. medRxiv preprint 2022 doi: https://doi.org/10.1101/2022.02.21.22271312
28. Schliemann D, Donnelly M, Dahlui M, et al. The 'Be Cancer Alert Campaign': protocol to evaluate a mass media campaign to raise awareness about breast and colorectal cancer in Malaysia. *BMC cancer* 2018;18(1):881.
29. Field A. Discovering statistics using IBM SPSS statistics. 4th ed. Los Angeles: Sage Publications 2013.
30. Nunnally JC, Bernstein IH. Psychometric theory. New York: McGraw-Hill Inc 1994.
31. Taber KS. The Use of Cronbach's Alpha When Developing and Reporting Research Instruments in Science Education. Res Sci Educ. 2018; 48, 1273–1296. https://doi.org/10.1007/s11165-016-9602-2
32. Al-Naggar RA, Al-Kubaisy W, Yap BW, et al. Attitudes towards colorectal cancer (CRC) and CRC screening tests among elderly Malay patients. *Asian Pac J Cancer P* 2015;16(2):667-74.
33. Yusoff HM, Daud N, Noor NM, et al. Participation and Barriers to Colorectal Cancer Screening in Malaysia. *Asian Pacific Journal of Cancer Prevention* 2012;13(8):3983-87. doi: 10.7314/apjcp.2012.13.8.3983
34. Tabachnick BG, Fidell LS. Using multivariate statistics. 6 ed. Boston, USA: Pearson 2007.
35. International Business Machines Corporation (IBM). SPSS Statistics, Factor Analysis Extraction. 2016. Accessed on 1 January 2022. Available: https://www.ibm.com/docs/en/spss-statistics/24.0.0?topic=analysis-factor-extraction
36. Allen M. The sage encyclopedia of communication research methods. Thousand Oaks, CA: SAGE Publications. 2017. doi: 10.4135/9781483381411
37. Htay MNN, Schliemann D, Dahlui M, et al. Validation of the Champion Health Belief Model Scale for an Investigation of Breast Cancer Screening Behaviour in Malaysia. *Int J Environ Res Public Health*. 2021;18(17):9311.
38. Ministry of Health Malaysia P. Management of Colorectal Carcinoma: Putrajaya, 2017.
39. Van Tze CN, Fitzgerald H, Qureshi A, et al. Understanding colorectal cancer in Malaysia: A mini-review and pioneering colorectal cancer awareness, screening and treatment project. 2017
40. Magaji BA, Moy FM, Roslani AC, et al. Survival rates and predictors of survival among colorectal cancer patients in a Malaysian tertiary hospital. *BMC cancer* 2017;17(1):339.
41. Lau J, Lim TZ, Wong GJ, Tan KK. The health belief model and colorectal cancer screening in the general population: A systematic review. *Prev Med Rep.* 2020; 20:101223. doi: 10.1016/j.pmedr.2020.101223
42. Lee SY, Lee EE, Rhee YS. Adaptation and validation of the health belief model scale for colorectal cancer screening. *Nurs Health Sci.* 2020;22:355–363.

For peer review only
